# Climate risks and adaptation strategies of farmers in East Africa and South Asia

**DOI:** 10.1038/s41598-021-89391-1

**Published:** 2021-05-18

**Authors:** Jeetendra Prakash Aryal, Tek Bahadur Sapkota, Dil Bahadur Rahut, Paswel Marenya, Clare M. Stirling

**Affiliations:** 1 International Maize and Wheat Improvement Centre (CIMMYT), Carretera México-Veracruz, Km. 45, El Batán, Texcoco, 56237 Mexico; 2 Asian Development Bank Institute (ADBI), Kasumigaseki Building 8F, 3-2-5 Kasumigaseki, Chiyoda-ku, Tokyo, 100-6008 Japan; 3 International Maize and Wheat Improvement Center (CIMMYT), Nairobi, Kenya; 4 Global R&D Technology Lead, Cocoa Life, Mondelez International, Birmingham, UK

**Keywords:** Climate sciences, Environmental social sciences, Natural hazards

## Abstract

Understanding major climate risks, adaptation strategies, and factors influencing the choice of those strategies is crucial to reduce farmers’ vulnerability. Employing comprehensive data from 2822 farm households in Ethiopia and Kenya (East Africa; EA) and 1902 farm households in Bangladesh, India, and Nepal (South Asia; SA), this study investigates the main climate risks that farmers faced and the adaptation strategies they used. Among others, excessive rainfall and heightened crop pest/disease incidence are commonly observed climate-induced risks in all study areas, while cyclones and salinity are unique to Bangladesh. Drought is prevalent in Ethiopia, India, Kenya, and Nepal. Farmers in those countries responded with strategies that include change in farming practices, sustainable land management, reduce consumption, sell assets, use savings and borrowings, seek alternative employment and assistance from government or NGO. In general, farmers faced several multiple climate risks simultaneously and they responded with multiple adaptation strategies. Therefore, this study used a multivariate probit (MVP) approach to examine the factors influencing the adoption of adaptation strategies. Unlike other studies, we also tested and corrected for possible endogeneity in model estimation. All the countries mentioned have low adaptive capacity to address climate change, which is further weakened by inadequate governance and inefficient institutions. We observed significant differences in the choice of adaptation strategies between male-headed households (MHHs) and female-headed households (FHHs), as well as across countries. Generally, MHHs are more likely to seek additional employment and change agricultural practices, while FHHs and households headed by older persons tend to reduce consumption and rely on savings and borrowings. Institutional support for adaptation is much less in EA compared to SA. Training on alternative farming practices, enhancing non-farm employment options, better institutional support, and social security for older farmers are crucial for climate change adaptation in both regions.

## Introduction

Climate-induced risks threaten nations worldwide, but poor countries are particularly vulnerable because of their low adaptive capacity and heavy reliance on climate-sensitive sectors such as agriculture^1,2^. Climate change has adversely affected the agricultural production in East Africa (EA) and South Asia (SA) and is expected to get worse in future^2–11^. It is projected that average temperatures in SA will increase by 0.5–1.2 °C by 2020, 0.88–3.16 °C by 2050, and 1.56–5.44 °C by 2080^12^. Several studies in EA show a negative impact of climate change on agricultural production and food security^3,13,14^. Similarly, the impacts of climate change on major cereal crops are usually negative in SA^4,15,16^. Climate change-induced heat-stress may convert the substantial proportion of the Indo-Gangetic Plains (IGP) of SA, a major food-basket of the region, into an unsuitable area for wheat production by 2050^17^.

Frequent occurrence of extreme heat events and increasing aridity will have several adverse repercussions on the entire agricultural system in EA^7,18^. Besides the direct risks of climate change, the resultant effects such as increased crop pests/diseases and livestock diseases are other major climate-induced risks facing the farmer in SA^19^ and EA^3^. For example, with global warming, yield loss of major crops—maize, wheat, and rice—due to insect pests will increase by 10 to 25% globally^20^.

In EA, climate risks are expected to be more severe in future as the temperature in this region is projected to rise faster than the rest of the world, which could exceed 2 °C by mid- twenty-first century and 4 °C by the end of the twenty-first century^21^. For instance, the drought of 2010–2011 resulted in massive crop failures in Ethiopia and Kenya, which raised the price of maize in Kenya by 246%^22^. In South Asia, there is a projection of 0.5–1.2 °C rise in temperature by 2020, 0.88–3.16 °C by 2050, and 1.56–5.44 °C by 2080^12^. Though the climate risks and vulnerability vary across countries in SA, the impacts of climate change will be quite severe in this region, even with a warming of 1.5–2 °C^11^. Data between 2001 and 2015 from SA revealed that flood risks are highly catastrophic in this region, particularly in the Ganges basin in North India, the Indus basin in Pakistan, and majority of Bangladesh^23^. In both regions, as the majority of farmers are smallholders, the impacts of climate change is pervasive due to a lack of resources and capacity to invest in adaptation. A detailed review of literature on climate risks and climate extreme events in the study countries is provided in Supplementary Material 1. In summary, the review shows that the region is vulnerable to climate and farmers experienced several climate risks such as drought, flooding and salination which varied in intensity across the region. Literature also shows that despite having low resilience capacity, the farm households in these region adopted several measure to cope with the climate risk.

Climate-induced risks are critical for both EA and SA as these regions represent the last frontiers of global poverty, with large population and recent economic development^15,24,25^. This makes adaptation critical to safeguard agricultural production and diminish the adverse impacts of climate change on farmers’ livelihoods^26–28^. Studies in both regions show that adoption of adaptation strategies depends on numerous factors, including economic, social, political, and institutional environments^2,3,29,30^. Access to institutional facilities such as timely support from extension services, farmer cooperatives, and government and non-governmental organizations, along with the knowledge of adaptation options, largely determines the choice of adaptation strategies^29,31^. Social factors such as gender can fundamentally govern the choice of adaptation strategies^32–35^. Therefore, a systematic understanding of how farmers are adapting and what factors affect their choice of adaptation strategies is crucial for future design of adaptation policies. To our knowledge, a primary data-based systematic study to understand the climatic risks experienced by smallholder farm households in EA and SA, available adaptation strategies, and factors affecting their choice is still scanty.

This study examines the key climate risks and the adaptation strategies applied to minimize the adverse impact of these risks on their livelihood by the farmers in EA and SA. We used rich datasets collected from 2287 farm households in Ethiopia, 535 in Kenya, 630 in Bangladesh, 641 in India, and 631 in Nepal. This study contributes to the current literature in three folds: (1) it is a comprehensive study of climate risks faced, and adaptation strategies adopted by the farmers in EA and SA; (2) it uses large datasets from EA and SA to carry out a comparative assessment of factors influencing the choice of adaptation strategies and (3) it provides not only the risk profiles of the study countries, but also assesses the macroeconomic factors limiting the national adaptation capacity and possible detrimental impacts of the key macroeconomic factors on the efficiency of national adaptation programs.

## Results

### Major climate risks and adaptation strategies

**Figure 1:**
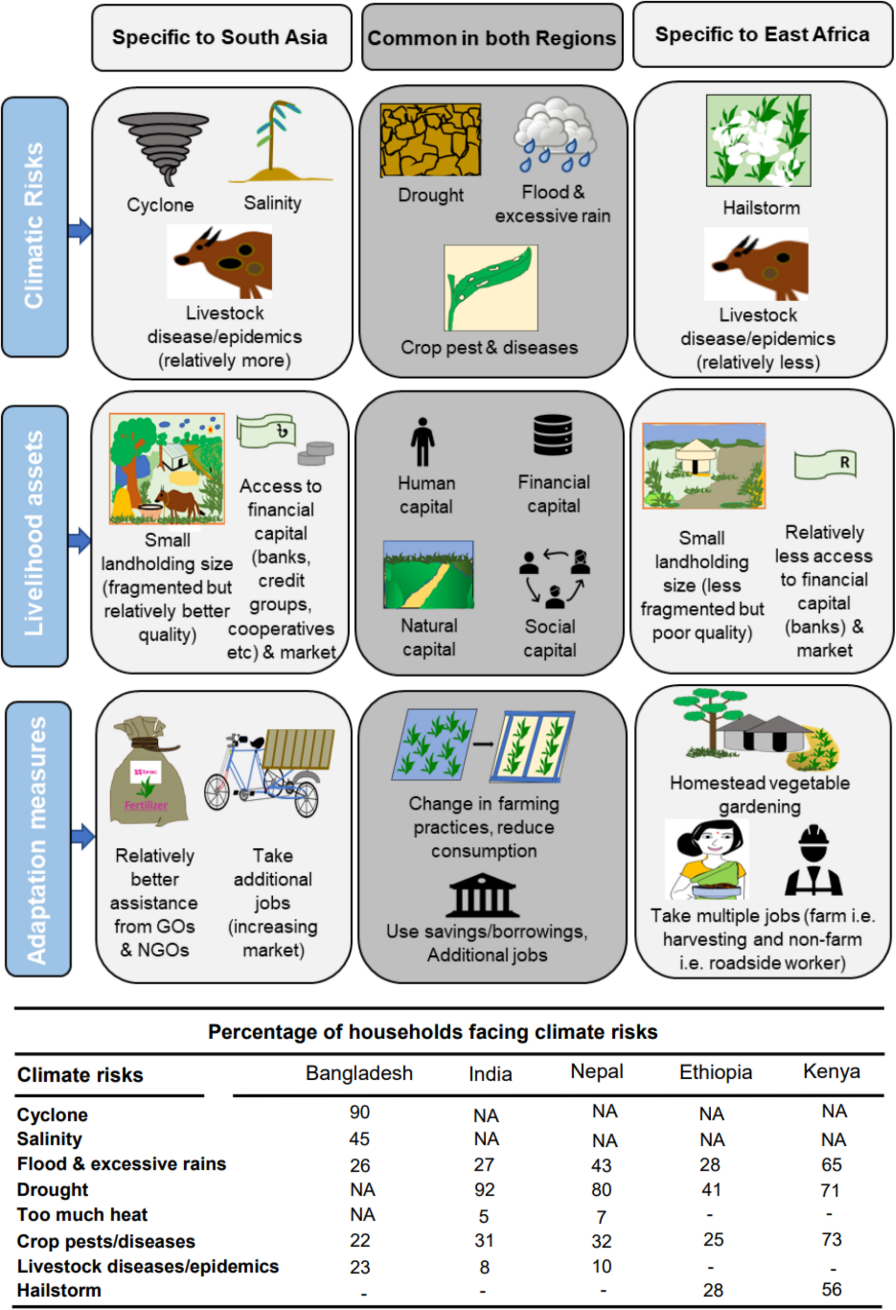
Major climate risks and adaptation strategies. Source: Prepared by the authors using Flowchart Maker and Online Diagram Software (https://app.diagrams.net/).

Farmers’ choice of adaptation strategies against climatic risks depends on the type of risk and available livelihood assets^36^ and adaptation options at their disposal. Therefore, we analyzed the major climatic risks, available livelihood assets, and major adaptation measures in all study sites in SA and EA (Fig. 1). The study sites in both regions experience recurrent drought stress induced primarily by higher temperature and short annual rainfall window leading to a prolonged dry period. Study sites in SA experience flooding and cropland inundation in summer, mainly due to excessive rainfall and flat topography. Similarly, study sites in EA also experience flooding due to few heavy rainfall events. Crop and livestock diseases constitute major threats to agriculture in both regions. Study sites in Bangladesh are affected by cyclones and cropland salinity due to sea-water intrusion, whereas those in EA suffer from hailstorms (Fig. 1). In Bangladesh, 90% of the sample households experienced cyclone during 2008–2012, 45% experienced salinity, and 26% experienced flood and extreme rain, while in India, 92% reported drought and 27% too much rains and flood, and in Nepal, 80% experienced drought and 43% flood and excessive rains (Fig. 1). In EA, none of the households experienced cyclone and salinity while 71% in Kenya and 41% in Ethiopia experienced drought and 64%, and 28% experienced excessive rainfall, respectively. Similarly, 56% and 28% of the households in Kenya and Ethiopia experienced hailstorms, and 73% and 25% of the households experienced crop pests/disease. It means the climate risks are location-specific: the coastal areas suffer from cyclone and salinity while the inland suffers from drought. Nevertheless, both coastal and interior land suffer from crop pest/disease and livestock diseases.

Average landholdings are relatively larger in EA than SA but the quality of farm land is better in SA. Farmers in EA have greater livestock assets, on average, whereas farmers in SA have better access to finance and markets (Fig. 1).

Households have applied seven types of strategies to adapt to climate risks (Table 1). In Nepal, the change in farming practices (40%) is the most pertinent strategy, followed by take additional jobs (16%), use past savings/borrowing (11%), and reduce consumption (6%). In Bangladesh and India, we found a similar pattern in climate adaptation: use of savings /borrowing was the most applied strategy (42% and 40%), followed by a change in farming practices (29% and 25%), reduce consumption (26% and 28%) and take up an additional job (22% and 21%). In Bangladesh, many farmers also obtained assistance from the government and NGOs to adapt to climate risks. In Ethiopia, use of saving and borrowing (21%) followed by a change in farming practices (17%) and reduce consumption (10%) were the major adaptation strategies, while in Kenya, 39% adopted change in farming practices followed by reduce consumption (34%), sustainable land management (12%) and use of saving and borrowing (11%).

**Table 1:** Major climate risks and coping strategies applied by farm households in the study area.

	Bangladesh	Bihar (India)		Nepal (Terai)		Ethiopia	Kenya	
	Percentage of households applying coping strategies							
Change in farming practices^1^ (Y1)	29		25		40	17		39
Use past savings/borrowing^2^ (Y2)	42		40		11	21		11
Reduce consumption^3^ (Y3)	26		28			10		34
Seek off-farm or other employment (Y4)	22		21		16	3		4
Take assistance from government (Y5)	24		7		5	4		0
Take assistance from NGO^5^ (Y6)	18		2		1	NA		0
Sustainable land management^6^ (Y7)	NA		NA		NA	0		12

NA and − refer to not applicable and no data, respectively. Multiple responses are possible.

1. Change in farming practices include replanting, use of chemical fertilizer, use of pesticide and herbicide, buy livestock replacement, and diversify crops, plots, and livestock. 2. Use past savings/borrowings also includes selling assets (i.e., livestock, land, and jewellery). We also included ‘buying food from the market’ within this as the farmer needs to either use past savings or the borrowed money to buy the food or buy food on loan. 3. Reduce consumption includes eating less (reducing meal quantity/quality) and spending less on non-food items. 4. Take additional jobs includes both in the agricultural and non-agricultural sector. 5. Includes help from self-help groups, local and other non-governmental organizations. 6. Sustainable land management includes soil and stone bunds, tree planting, used soil and water conservation, and using a ridge.

### Factors influencing the choice of adaptation strategies

To identify factors affecting the choice of adaptation strategies by the farmers, we used a multivariate probit model (MVP). Farmers have adopted different strategies concurrently which increases the likelihood that the choices are correlated and requires the pair-wise testing of correlation coefficients. We tested the pair-wise correlation coefficients across the residuals of MVP models for all five countries in this study and found many statistically significant correlations (Supplementary Material 2), supporting our hypothesis that adaptation decisions are correlated. The likelihood ratio tests [for Bangladesh: chi2(15) = 188.24; Prob > chi2 = 0.0000; for Bihar (India): chi2(10) = 148.51; Prob > chi2 = 0.0000; for Terai (Nepal): chi2(10) = 108.23; Prob > chi2 = 0.0000; for Ethiopia: chi2(10) = 99.11; Prob > chi2 = 0.0000; for Kenya: chi2(10) = 83.20; Prob > chi2 = 0.0000] for all five countries rejected the null hypothesis that the covariance of error terms across equations are not correlated. This justifies the rationale for using an MVP rather than the probit model for individual strategies. Another important finding is that farmers consider some coping strategies as complementary while others as substitutions, but it varies across the countries.

Table 2 presents the results of the MVP models estimated using maximum likelihood methods for all countries under study. Wald Chi-square tests for each of the models estimated are significant at 1% level, indicating that the model specification is valid and the explanatory variables relevant for explaining farmers’ decisions to apply the respective adaptation strategy. The description and the summary statistics of the variable, including the dummy variables used in the econometric analysis, is presented in Supplementary Material 3.

**Table 2:** Choice of multiple risk coping strategies by farmers in Bangladesh, India, Nepal, Ethiopia and Kenya.

	Y1	Y2	Y3	Y4	Y5	Y6	Y7
	Y1 = Change in farming practices	Y2 = Use of savings and borrowing	Y3 = reduce consumption	Y4 = Seek additional jobs	Y5 = Take assistance from NGOs (a)	Y6 = Take assistance from government	Y7 = Sustainable land management
**Risk coping strategies used by farmers in Bangladesh**							
Male headed HH	0.237** (0.093)	− 0.322 (0.321)	0.282*** (0.097)	0.211** (0.085)	0.317*** (0.113)	− 0.275*** (0.103)	
Age	− 0.007 (0.005)	0.012** (0.006)	0.021*** (0.006)	− 0.006** (0.003)	0.002 (0.006)	0.005 (0.006)	
Occup in Ag	0.471*** (0.103)	0.173*** (0.052)	− 0.158*** (0.049)	0.304 (0.312)	0.299 (0.302)	0.211*** (0.064)	
Edu: Primary	− 0.088 (0.163)	0.161*** (0.051)	0.201 (0.193)	− 0.073 (0.154)	− 0.203 (0.196)	− 0.170 (0.184)	
Edu: Secondary	0.243** (0.118)	− 0.101 (0.161)	0.185* (0.098)	− 0.357** (0.168)	− 0.171 (0.183)	0.158* (0.083)	
Edu: Higher secondary & above	− 0.048 (0.276)	− 0.303 (0.275)	− 0.477* (0.262)	0.435*** (0.109)	0.187** (0.091)	0.170*** (0.036)	
Labor	0.045 (0.052)	0.061 (0.059)	0.129** (0.059)	0.189*** (0.056)	− 0.044 (0.053)	− 0.054 (0.060)	
Land	0.276** (0.135)	0.296** (0.147)	0.156 (0.150)	− 0.301* (0.165)	− 0.235 (0.229)	− 0.531*** (0.187)	
Livestock (TLU)	0.115** (0.055)	0.127** (0.051)	− 0.116* (0.068)	− 0.182*** (0.053)	− 0.107 (0.086)	− 0.064 (0.080)	
Assets	0.113 (0.136)	0.195*** (0.063)	− 0.151*** (0.046)	− 0.201** (0.098)	0.287*** (0.109)	− 0.183** (0.081)	
Food secure HH	0.100** (0.047)	0.302** (0.147)	− 0.251** (0.110)	− 0.197 (0.147)	0.098 (0.172)	0.073 (0.169)	
Training	0.435** (0.201)	0.305 (0.299)	− 0.317 (0.308)	0.254** (0.127)	0.198 (0.215)	0.275 (0.266)	
Credit	0.105 (0.133)	0.327** (0.152)	− 0.171 (0.167)	− 0.140 (0.153)	0.307*** (0.103)	− 0.269 (0.178)	
Member	0.197* (0.102)	0.171** (0.083)	0.144 (0.151)	0.148 (0.147)	0.236* (0.125)	0.261** (0.122)	
Market	0.001 (0.015)	0.011 (0.014)	− 0.042* (0.023)	0.130*** (0.029)	0.032** (0.015)	0.029** (0.014)	
Extension	0.084*** (0.026)	0.025 (0.024)	− 0.051** (0.024)	− 0.025 (0.023)	0.036** (0.017)	0.047*** (0.013)	
Constant	− 1.053** (0.419)	− 1.815*** (0.446)	− 1.588*** (0.481)	− 1.476*** (0.473)	− 2.201*** (0.554)	− 1.452*** (0.469)	
**Risk coping strategies used by farmers in Bihar (India)**							
Male headed HH	0.321*** (0.113)	− 0.208 (0.250)	0.364*** (0.122)	0.356 (0.360)	1.427* (0.793)		
Age	0.006 (0.007)	− 0.007 (0.008)	0.017*** (0.006)	− 0.023** (0.011)	0.010 (0.012)		
Occup in Agri	0.354*** (0.125)	0.251*** (0.089)	0.403** (0.195)	0.195* (0.103)	− 0.311 (0.374)		
Edu: Primary	− 0.165 (0.212)	0.303*** (0.109)	0.151 (0.232)	− 0.174 (0.199)	0.217 (0.301)		
Edu: Secondary	0.315** (0.152)	0.134 (0.148)	− 0.235** (0.102)	0.307** (0.153)	0.217 (0.280)		
Edu: Higher	− 0.289*** (0.091)	0.273*** (0.085)	− 0.250** (0.109)	0.419** (0.201)	0.258** (0.105)		
Labor	0.408*** (0.142)	0.443*** (0.165)	− 0.428 (0.415)	0.466*** (0.152)	− 0.811 (1.078)		
Land	0.309*** (0.110)	− 0.184 (0.192)	− 0.207*** (0.072)	− 0.195 (0.193)	0.157** (0.065)		
Livestock	0.103 (0.081)	0.115** (0.051)	− 0.298*** (0.100)	0.085 (0.087)	− 0.151 (0.170)		
Asset	0.166 (0.161)	0.128** (0.056)	0.119 (0.113)	0.121*** (0.029)	0.167 (0.221)		
Food secure	− 0.113 (0.119)	0.135 (0.151)	− 0.351*** (0.127)	− 0.265* (0.137)	− 0.233 (0.242)		
Training	0.304*** (0.102)	0.093 (0.115)	0.391*** (0.129)	0.407*** (0.133)	0.191 (0.213)		
Credit	0.305** (0.125)	0.442*** (0.127)	0.265** (0.130)	0.285** (0.129)	− 0.209 (0.247)		
Membership	0.394* (0.205)	0.265* (0.145)	− 0.320** (0.159)	− 0.275 (0.251)	0.161** (0.075)		
Market	− 0.042** (0.020)	− 0.087*** (0.021)	0.026* (0.014)	0.010 (0.012)	0.039** (0.019)		
Extension	0.056*** (0.014)	− 0.067*** (0.019)	0.091*** (0.021)	0.027 (0.034)	0.041** (0.018)		
Constant	− 1.312*** (0.345)	− 1.417*** (0.469)	− 2.210*** (0.653)	− 0.983** (0.389)	− 2.315** (1.102)		
**Risk coping strategies used by farmers in Terai (Nepal)**							
Male-headed HH	0.262*** (0.066)	− 0.417** (0.172)	− 0.228 (0.208)	0.533*** (0.190)	0.615** (0.259)		
Age of HH head	− 0.003 (0.004)	− 0.000 (0.006)	− 0.004 (0.005)	− 0.015** (0.007)	− 0.005 (0.008)		
Occup in Agri	0.323*** (0.077)	0.245** (0.108)	0.198*** (0.053)	− 0.135 (0.186)	− 0.232 (0.234)		
Edu: Primary	0.167 (0.150)	0.196 (0.203)	0.295*** (0.098)	− 0.166 (0.215)	− 0.109 (0.313)		
Edu: Secondary	0.281*** (0.103)	0.250** (0.121)	0.201 (0.223)	0.310** (0.146)	− 0.223 (0.305)		
Edu: Higher secondary & above	− 0.191*** (0.061)	0.242*** (0.079)	− 0.402*** (0.133)	0.376*** (0.115)	0.161* (0.086)		
Labor	0.103*** (0.030)	− 0.039 (0.054)	0.031 (0.051)	− 0.131*** (0.041)	0.053 (0.061)		
Land	0.153** (0.067)	− 0.311*** (0.097)	− 1.560*** (0.355)	0.085 (0.082)	− 0.102 (0.113)		
Livestock (TLU)	0.174*** (0.041)	0.243*** (0.050)	− 0.085 (0.091)	− 0.113 (0.092)	0.058 (0.073)		
Asset	0.251*** (0.078)	− 0.396*** (0.101)	− 0.382** (0.181)	0.173** (0.086)	0.088 (0.100)		
Food secure HH	0.098*** (0.029)	− 0.035 (0.204)	− 0.276*** (0.068)	− 0.153 (0.231)	0.358 (0.433)		
Training	0.363*** (0.119)	0.405 (0.417)	− 0.119* (0.062)	0.133** (0.057)	− 0.284 (0.301)		
Credit	− 0.144* (0.078)	0.225** (0.105)	0.163 (0.171)	− 0.252*** (0.087)	− 0.157 (0.209)		
Membership	0.250*** (0.093)	0.296** (0.144)	− 0.158 (0.163)	0.161** (0.075)	0.213** (0.103)		
Market	0.053*** (0.015)	− 0.042 (0.055)	0.025 (0.037)	0.065*** (0.013)	− 0.196*** (0.048)		
Extension	0.045*** (0.013)	− 0.017 (0.019)	0.043 (0.058)	0.046** (0.022)	0.068** (0.030)		
Constant	− 0.978*** (0.356)	− 1.314*** (0.392)	− 1.311*** (0.471)	− 3.085*** (0.722)	− 1.576*** (0.513)		
**Risk coping strategies used by farmers in Ethiopia**							
Male-headed HH	0.155*** (0.037)	0.149*** (0.042)	− 0.161*** (0.059)	0.291*** (0.074)	0.247** (0.103)		
Age of HH head	− 0.002 (0.003)	− 0.005* (0.003)	− 0.002 (0.003)	− 0.010** (0.005)	− 0.005 (0.004)		
Occup in Agri	0.190*** (0.051)	0.111 (0.149)	0.145** (0.071)	− 0.144 (0.238)	0.318 (0.275)		
Edu: Primary	0.033 (0.077)	− 0.260*** (0.073)	− 0.068 (0.089)	− 0.207 (0.128)	0.065 (0.117)		
Edu: Secondary	0.082 (0.091)	− 0.332*** (0.089)	− 0.118** (0.051)	0.314* (0.161)	0.154* (0.084)		
Edu: Higher secondary & above	0.303** (0.121)	− 0.335* (0.177)	− 0.215*** (0.067)	0.270*** (0.091)	0.356*** (0.130)		
Labor	0.078*** (0.016)	0.056*** (0.016)	0.045** (0.019)	0.125*** (0.028)	− 0.012 (0.026)		
Land	0.112*** (0.027)	0.114*** (0.017)	− 0.152*** (0.022)	− 0.031 (0.039)	− 0.061* (0.036)		
Livestock (TLU)	0.122*** (0.038)	0.015* (0.008)	− 0.035*** (0.013)	− 0.084*** (0.016)	0.019 (0.012)		
Asset	0.271*** (0.075)	− 0.135*** (0.043)	− 0.091** (0.042)	− 0.065** (0.031)	− 0.000 (0.001)		
Food secure HH	− 0.121* (0.069)	− 0.251*** (0.067)	− 0.289*** (0.082)	− 0.215* (0.119)	− 0.076 (0.106)		
Training	0.188*** (0.052)	0.314** (0.137)	− 0.074 (0.142)	− 0.236 (0.190)	0.370 (0.256)		
Credit	0.157** (0.068)	0.140** (0.066)	− 0.002 (0.080)	− 0.079 (0.120)	− 0.136 (0.108)		
Membership	− 0.096*** (0.031)	0.001* (0.000)	− 0.016*** (0.005)	0.001 (0.001)	0.002** (0.001)		
Market	0.013*** (0.003)	− 0.000 (0.001)	− 0.002*** (0.001)	− 0.063*** (0.021)	0.013** (0.006)		
Extension	0.021** (0.010)	0.125 (0.127)	0.101 (0.121)	0.091 (0.232)	0.247 (0.264)		
Constant	− 0.623*** (0.226)	− 1.244*** (0.239)	− 1.225*** (0.271)	− 0.859** (0.370)	− 2.221*** (0.441)		
**Risk coping strategies used by farmers in Kenya**							
Male-headed HH	0.338** (0.168)	0.153** (0.074)	− 0.136** (0.060)	0.286*** (0.107)			0.309** (0.122)
Age of HH head	− 0.008* (0.005)	0.005 (0.006)	0.014*** (0.005)	− 0.048** (0.019)			− 0.011* (0.006)
Occup. in Agri	0.139*** (0.051)	0.156 (0.187)	0.120** (0.056)	− 0.113 (0.456)			0.187*** (0.070)
Edu: Primary	0.237 (0.282)	0.136** (0.061)	− 0.303 (0.256)	− 0.649 (0.596)			− 0.300 (0.297)
Edu: Secondary	0.273** (0.110)	0.070 (0.323)	− 0.155 (0.247)	0.128* (0.074)			− 0.455 (0.291)
Edu: Higher secondary & above	− 0.113* (0.065)	− 0.357*** (0.104)	− 0.237*** (0.072)	0.415*** (0.151)			0.315*** (0.103)
Labor	0.138*** (0.025)	0.136*** (0.034)	0.052** (0.025)	0.368*** (0.073)			0.021 (0.032)
Land	0.185*** (0.058)	0.183** (0.086)	− 0.155*** (0.047)	0.083 (0.097)			0.137** (0.061)
Livestock (TLU)	0.117*** (0.036)	− 0.111* (0.062)	− 0.103*** (0.034)	− 0.115** (0.046)			0.009 (0.044)
Asset	0.161** (0.075)	− 0.045 (0.051)	0.036 (0.035)	0.204*** (0.054)			− 0.123** (0.050)
Food secure HH	− 0.133* (0.072)	− 0.149** (0.067)	− 0.174*** (0.064)	− 0.669 (0.525)			− 0.300* (0.175)
Training	0.400*** (0.147)	0.282 (0.199)	− 0.211 (0.140)	0.238** (0.095)			0.143** (0.059)
Credit	0.148** (0.069)	0.221*** (0.062)	− 0.147*** (0.053)	0.023 (0.542)			− 0.011 (0.196)
Membership	0.181* (0.101)	− 0.204 (0.215)	0.028 (0.148)	0.272* (0.155)			0.221*** (0.073)
Market	0.338** (0.168)	0.153*** (0.044)	0.136** (0.060)	− 0.062*** (0.023)			0.409* (0.222)
Extension	0.078** (0.035)	0.005 (0.006)	0.051 (0.067)	− 0.008 (0.019)			0.048*** (0.010)
Constant	− 1.237*** (0.332)	− 1.313** (0.536)	− 2.081*** (0.478)	− 1.479*** (0.493)			− 1.253*** (0.395)

For Bangladesh: Log likelihood = − 1912.31; Wald chi-square (96) = 163.14; Prob > Chi-square = 0.000.

For Bihar (India): Log likelihood = − 1187.69; Wald Chi-square (80) = 151.23; Prob > Chi-square = 0.000.

For Terai (Nepal): Log likelihood = − 1001.64; Wald Chi-square (80) = 162.95; Prob > Chi-square = 0.000.

For Ethiopia: Log likelihood = − 1001.64; Wald Chi-square (80) = 162.95; Prob > Chi-square = 0.000.

For Kenya: Log likelihood = − 1001.64; Wald Chi-square (80) = 162.95; Prob > Chi-square = 0.000.

*Y1* change in farming practices, *Y2* use of savings and borrowing, *Y3* reduce consumption, *Y4* seek additional jobs, *Y5* take assistance from government (for India and Nepal it refers to taking assistances from both governmental and/or non-governmental organizations), *Y6* take assistance from non-governmental organizations, *Y7* Sustainable land management.

*, **, ***Refer to 10, 5 and 1% level of significance, respectively. Standard errors are in parentheses.

#### Gender and climate change adaptation

The choice of adaptation strategies varies greatly by the gender of the household head (Table 2). In all countries, MHHs are more likely than FHHs to apply multiple adaptation strategies, yet the types of strategies vary across countries. In Bangladesh, MHHs are more likely to change farming practices, reduce consumption, seek additional employment and support from the government for adaptation, while in Bihar, they change farming practices, reduce consumption, and also seek help from the government. In Nepal, MHHs are more likely to change farming practices, seek an additional job and seek help from government institutions to address climate risks. In Ethiopia, MHHs are more likely to change farming practices, use savings and borrowing, seek additional employment and assistance from the government or NGOs, but do not reduce consumption. In Kenya, MHHs are also more likely to change farming practices, use savings and borrowing, seek additional employment and apply sustainable land management practices for adaptation, but less likely to reduce consumption.

In both SA and EA, MHHs are more likely to change their farming practices to adapt to climate change. MHHs in both regions tend to seek additional employment, except in Bihar. Though the coefficient for seeking additional employment was positive, it was insignificant, which may be due to the fact that Bihar is a poor state with limited off-farm employment. MHHs in EA are more likely to use saving and borrowing as adaptation strategies, while in Nepal they are less likely to do so. In India and Bangladesh, there is no significant difference between MHHs and FHHs regarding the use of savings and borrowing as adaptation strategies.

#### Age and climate change adaptation

In Bangladesh, India, and Kenya, the coefficient of age is positive and highly significant for reduced consumption, indicating that the households with older heads are more likely to reduce consumption as an adaptation strategy. Older people generally have low income and, when they encounter shocks, they do not apply adaptation strategies that require more resources but rather tend to reduce consumption. The coefficient of the age of the household head is negative for seeking additional employment in both SA and EA, indicating that older individuals either lack required skills or the stamina to perform an additional job; hence they are less likely to seek an additional job for adapting to climate risks. In Bangladesh and Ethiopia, older individuals are also more likely to use their savings and borrowing for adaptation. In Kenya, older individuals are less likely to change in farming practices and adopt sustainable land management. For older farmers, change in farming practices becomes less feasible and, thus, they need social security. This has important implications for public policy on how to provide social security for an aging rural population under increasing climate risks.

#### Livelihood and climate change adaptation

In both SA and EA, households whose main occupation is agriculture are more likely to change farming practices as an adaptation strategy. Since agriculture is the main source of income for such households, they tend to adopt strategies around agriculture. In Bangladesh, households whose main occupation is agriculture are more likely to seek support from NGOs and reduce consumption to adapt, while in Kenya they adopt sustainable land management practices.

#### Education and climate change adaptation

In all the countries studied, households whose head has a higher level of education are more likely to seek up an additional job as a strategy for climate adaptation. Households with a better level of education possess skills and knowledge, which enable them to take up additional jobs. Another important finding is that they do not reduce consumption for climate adaptation. This may be because they are able to adopt other options to adapt. In Bangladesh, Nepal, and Ethiopia, households with better-educated heads are also more likely to seek government and NGO support, as they have knowledge and awareness about such possibilities. In Kenya, the better-educated households are also more likely to adopt sustainable land management practices, because of their knowledge and understanding of such practices and the ability to learn.

#### Family labour and climate change adaptation

In all countries except Nepal, the coefficient of the household labour force is positive and highly significant for seeking an additional job, indicating that households with more labour capacity are more likely to seek additional jobs to adapt to climate risks. This is plausible because they are able to reallocate surplus labour to additional jobs and better adapt to climate risks.

In all countries except Bangladesh, the coefficient of household labour force is positively and significantly associated with a change in farming practice. Households with more labour are able to apply it to change the farming practice. In EA, the family labour capacity was positively associated with all adaptation strategies except seeking government support or applying sustainable land management practices.

#### Land ownership and climate change adaptation

Land size is positively associated with a change in farming practices in all countries studied, indicating that larger-scale farmers are more likely to change farming practices for climate change adaptation. In India, Nepal, and Kenya, larger-scale farmers are less likely to reduce consumption when faced with climate risks. Larger-scale farmers in Bangladesh and Ethiopia are more likely to use savings and borrowing, while it is opposite in Nepal.

#### Livestock ownership and climate change adaptation

In Bangladesh, India, Nepal, and Ethiopia, livestock ownership is positively associated with the use of saving and borrowing as adaptation strategies. Compared to other household assets such as land, livestock is easier to sell and thus, plays a vital role during times of economic stress^37^. Similarly, in Bangladesh, India, Ethiopia, and Kenya, livestock ownership is inversely associated with reducing consumption, highlighting the fact that households with more livestock are able to use this asset to minimize vulnerability to climate risks and do not have to reduce their consumption. In Bangladesh, Nepal, Ethiopia, and Kenya, households with more livestock are more likely to change farming practices for adaptation. In Ethiopia, Kenya, and Bangladesh, households with more livestock are less likely to seek additional employment.

#### Household assets and climate change adaptation

In India, Nepal, and Kenya, asset-rich households are more likely to seek additional employment for climate adaptation, whereas in Bangladesh and Ethiopia they are less likely to seek additional employment. In Bangladesh, Nepal, and Ethiopia, asset-rich households are less likely to reduce consumption to adapt to climate risks, and this may be because they are wealthier and have the capacity to absorb climate risk. Asset-rich households in Bangladesh and India are more likely to use savings and borrowing for climate adaptation, while such households in Nepal and Ethiopia are less likely to follow those strategies. Asset-rich households in Nepal, Ethiopia, and Kenya are more likely to change farming practices for adaptation.

#### Food security and climate change adaptation

As expected, food-secure households in all countries under study (except Nepal) are less likely to reduce consumption. To adapt to climatic risks, food-secure households in Bangladesh and Nepal are more likely to change farming practices, whereas this is not the case in Ethiopia and Kenya. In Bangladesh, food-secure households are more likely to use savings in response to climate risks; households in Ethiopia and Kenya are less likely to do so.

#### Training and climate change adaptation

Our results show that farmers who have received training are more likely to change farming practices as an adaptation strategy to climate risks in all locations, likely because they are more aware of farming practices to cope with climate risks; hence they might be expected to do so for adaptation. Households in Bangladesh, India, Nepal, and Kenya more likely to seek additional employment as a climate adaptation strategy, possibly due to networks they have established during training events.

#### Credit and climate change adaptation

In all locations, households with access to credit are more likely to use savings and borrowing as adaptation strategies. Households with access to credit in India, Ethiopia, and Kenya also change farming practices to adapt to climate risks while, in Nepal, households with access to credit are less likely to change farming practices.

#### Membership in village organization and climate change adaptation

Membership in an organization empowers farmers with contacts and sources of knowledge sharing regarding farm practices and strategies to adapt to climate risks. Our results show that farmers in SA and EA who belong to village organizations are more likely to change farming practices to adapt to climate risks. In Bangladesh, India, Nepal, and Ethiopia, such farmers are also more likely to seek government and NGO support, likely due to their enhanced networks and knowledge about approaching those entities. In India, Nepal, and Ethiopia, farmers who belong to village organizations are more likely to use saving and borrowings for adapting to climate risk. In Nepal and Kenya, we also found that farmers with membership in village organizations are more likely to seek additional employment, as an adaptation strategy.

#### Access to market and extension services and climate change adaptation

Access to markets and extension services is crucial for farmers to obtain inputs and information and sell their harvest, thus playing an important role in technology adoption. In Bangladesh, India, and Ethiopia, households that are further away from the market tend to seek support from the government and NGOs to adapt to climate change risks, while in Nepal, this is not the case. In Bangladesh, Nepal, Ethiopia, and Kenya, households that are further away from market seek additional employment as an adaptation to climate change risk. Households further away from the market in Nepal, Ethiopia, and Kenya mostly change farming practices to adapt to climate change, while this is not the case in India. Generally, the households that are further away from extension services are less likely to change farming practices.

## Discussion and policy implications

### Major climate risks and adaptation strategies

Countries with higher adaptive capacity are less vulnerable to climate change, irrespective of the level of exposure to climate risks^38–40^. Poverty, access to and ownership of economic resources, and human capital largely influence households’ choices of climate risk adaptation strategies^40–43^. Similarly, the capacity to adapt to climate change at the national level is associated with multiple factors, including socio-economic, political, and institutional quality^44,45^, institutional improvement such as good governance^46–48^. The countries in this study experience different levels of exposure to climate risks and have a differential capacity to adapt to climate change; hence the level of vulnerability varies across the countries (Supplementary Material 4).

Our findings show that many farm households in EA and SA experienced multiple climate risks, including drought, floods, and crop pests and diseases. Even though agriculture is the major sector supporting the livelihoods of most farm households in both regions, farmers adopt diversified adaptation measures, and this constitutes valuable intelligence to design policies and adaptation programs to support farm households and reduce their vulnerability to climate risks.

### Gender and social dimensions

In all countries we found a differential effect of gender in climate change adaptation, corroborating findings of other studies in EA^33,35,48–50^ and SA^41,51–53^. Female-headed households (FHHs) in both regions are more likely to pursue reduced consumption, as an adaptation strategy to climate change risks, and generally do not seek alternative employment. This might be related to the social-cultural issues which need to be studied further. Despite their increasing role in agriculture, females still have a lower capacity to adapt to climate change in both regions^54–56^.

### Institutional support, institutional quality, and livelihood diversification

In both SA and EA, institutional support to farmers for livelihood diversification is still inadequate, though such supports are gaining importance recently^57^. Institutional supports and their modus operandi also vary considerably across the countries. For example, our results show that non-governmental organizations are very active in supporting climate change risk adaptation by resource-poor farmers in Bangladesh, but not in other countries. India has programs that support such farmers in times of crisis. Yet, in rural areas of both EA and SA, livelihood options are limited and the efficiency of public institutions in reaching poor farmers is low, so increasing climate risks can severely increase their vulnerability^58^.

In line with the findings of other studies^41,59^, this study shows that households with non-farm income are more likely to apply adaptation measures, indicating that livelihood diversification can enhance adaptation. Therefore, promoting social protection programs that help diversify the livelihood options of smallholder farmers can enhance their adoption of adaptation strategies that reduce their risks from climate change^59^.

### Demographic differences in climate change adaptation

In both regions, elderly household heads are less likely to pursue alternative employment opportunities to adapt to climate risks. Changing skill required for alternative employment and increasing health risks with age are possible reasons^60,61^ but, this is outside the scope of our study and the topic requires further research. In any case, our observation suggests the need for social safety net programs to address differing levels of vulnerability across demographic groups.

### Wealth, access to credit, and market

Most farmers in both regions are poor in physical capital, such as land, livestock, and other household assets. Relatively wealthy households (in terms of land and livestock) are less likely to reduce consumption as a climate change adaptation whilst they prefer to either change in farming practices or use their savings and borrowings. Many previous studies have similar findings^26,62^. Another crucial finding is that access to credit and markets is an important influence on choices of climate adaptation strategies^5,63^.

### Policy implications

As indicated by the climate risk index score, the level of exposure to climate risk is extremely high in SA and EA, which calls for establishing the climate risk early warning system to prepare farmers in advance for likely climate risk. Agriculture is the major source of livelihoods and contributes significantly to national economies in both regions; at the same time, it is the sector that is most vulnerable to climate risks. It is crucial that governments in these countries invest in technology and strategies to reduce farmers' exposure and vulnerability. As these countries suffer from low GDP per capita, high level of poverty, high debt, and poor governance, it limits the capacity of the government to make an investment in managing and coping with the climate risk. Thus, governments should invest substantively in improving the economy, transparency, and governance and reducing poverty and inequality to enhance the national and household-level capacity in rural areas to cope with climate risk, which is likely to increase in coming years.

The micro/household level analysis shows that households in these regions are poor and have limited capacity to cope with the climate shocks on the one hand and on the other hand, have limited access to good institutions, which is central in defining the household’s resilience. Therefore, the government should initiate a policy to improve the household resilience capacity through livelihood diversification, improving awareness and access to sustainable Lang management practices, resilient seeds, and other farm imports and, finally, improving social safety nets.

Although both EA and SA are vulnerable and have low resilience, SA is better than EA for all indicators, suggesting the need for a more aggressive approach to improve resilience capacity in EA.

## Conclusion

Farmers in both EA and SA are facing several climatic risks. Cyclones, salinity, and floods, and excessive rainfall are the major climatic risks threatening farmers in Bangladesh, but drought is the major climate risk followed by flood and excessive rains in India, Nepal, Ethiopia, and Kenya. Crop/livestock pests and diseases are two major indirect effects of climate change in all countries under study.

Farmers adopt various adaptation strategies to minimize the effects of those risks on their livelihoods. Changes in farming practices is the most common adaptation strategy among farmers in Nepal and Kenya, whereas past savings and borrowing is a common adaptation strategy in Bangladesh, India, and Ethiopia. Unlike farmers in the other countries in this study, many in Bangladesh received government and NGO assistance.

Various factors determine the choices of climate change adaptation strategies by farmers in SA and EA. Gender plays an important role, and particularly the limited adaptive capacity of FHHs. MHHs are more likely to seek employment and change agricultural practices, while FHHs prefer to reduce consumption and rely on savings and borrowings. Therefore, enhancing gender equity, especially women’s access to off-farm jobs, can help them to better adapt to climate risks. Likewise, older farmers rely more on reducing consumption, rather than taking additional jobs, to adapt to climatic risks. This may be due to their health problems, suggesting the critical need for additional government support to older farmers, irrespective of their gender.

Household assets index, farmland, and livestock holdings also influence the farm household’s choice of adaptation strategy. Households with large farms mostly change farming practices to adapt to climatic risks. Those with more livestock rely on savings and borrowing as an adaptation strategy, because livestock act as an insurance mechanism for them.

Households with higher education rely mostly on additional jobs and also seek government and NGO help. They do not reduce consumption as an adaptation strategy. Similarly, farmers with training in farming and climate-related issues change farming practices and take additional jobs, as adaptations to climate change risk.

## Material and methods

### Data and sampling methods

**Figure 2:**
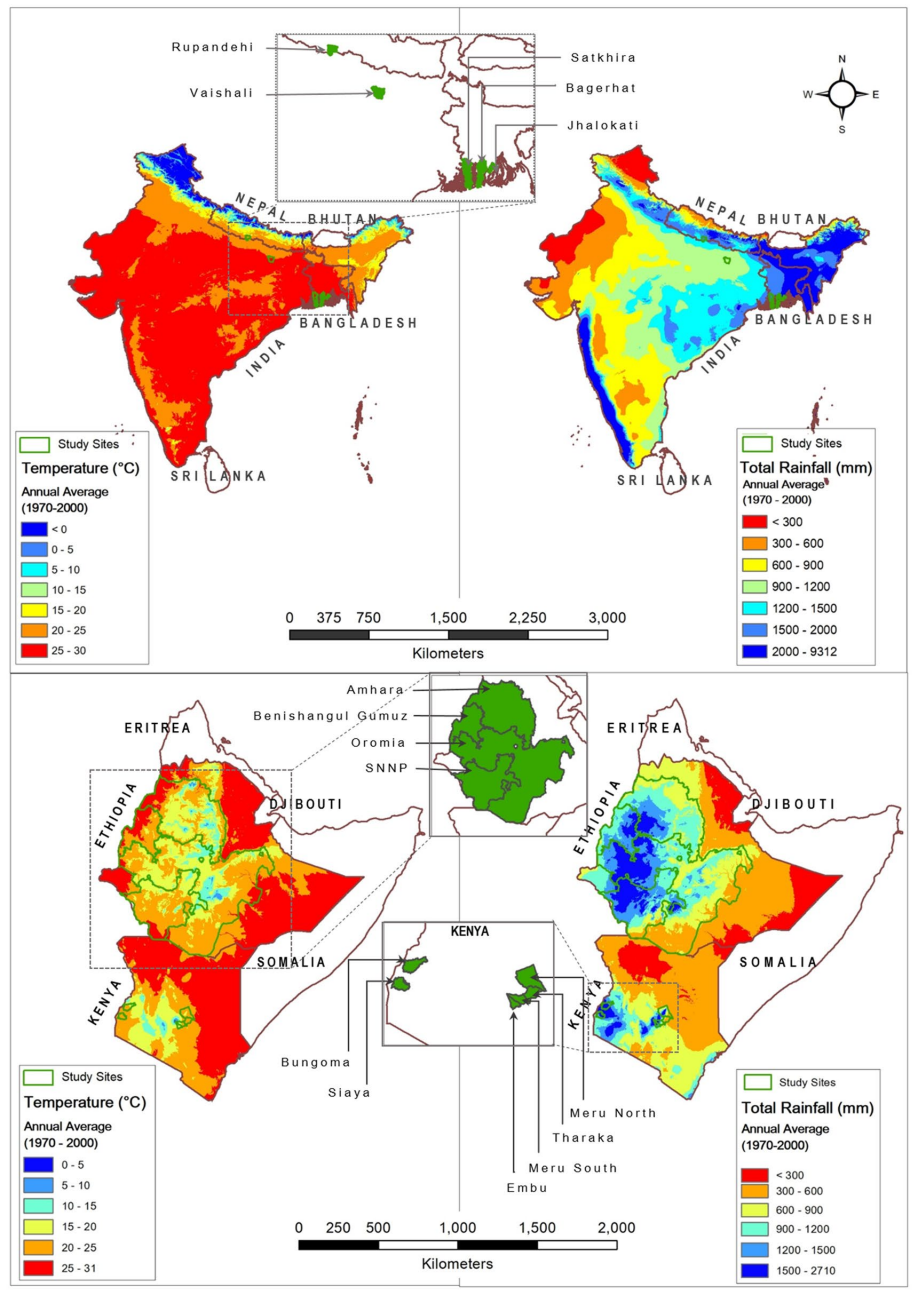
Map showing the study area along with temperature and rainfall. Source: Prepared by the authors using ArcGIS, version 10.8 (https://www.arcgis.com/index.html).

This research is based on a primary dataset collected in 2013 from a survey of 2822 households from EA (Ethiopia and Kenya) and 1902 households in SA (Bangladesh, India, and Nepal). Figure 2 shows the study area across five study countries in two continents together with their long-term rainfall and temperatures. Study sites in SA experience heat stress due to extremely high temperatures during most part of the year (Fig. 2). Extremely high temperatures in autumn reduce the tillering of winter crops during the early growth stage and result in terminal heat and early maturity in spring, all reducing grain yield. All study sites in SA also suffer from cropland inundation and flooding due to heavy rainfall during summer and from drought due to no or minimal rainfall from autumn to spring. Study sites in coastal Bangladesh also suffer from cropland inundation and salinization due to sea-water intrusion. The study sites in EA, on the other hand, have relatively milder temperatures but receive less precipitation, resulting in chronic drought stress (Fig. 2).

We used a multistage sampling procedure was used to select the broader regions (and agro-ecologies) for EA. The aim was to cover two contrasting agro-ecologies in both Ethiopia and Kenya. In terms of production, these were mid-altitude transitional and sub-humid areas where smallholder production was dominant. In Ethiopia, in the first stage, four regions (Amhara, Oromia, SNNP, and Benshangul) were selected and, in the second stage, we randomly selected districts (*woredas*) to include areas where maize and legume production were a major economic activity. Finally, at the village (*kebele*) level, households were randomly selected to reflect to participate in the surveys. The assignment of the number of households per number of kebeles per woreda was based on the most recent population census in each woreda so as to achieve proportional probability sampling. A similar process was used in Kenya: we selected five districts for the survey: two counties from the western region (Bungoma and Siaya) and three from the eastern region (Embu, Meru South, and Imenti South). These next level of sampling was sub-counties, sub-locations, and villages with the number of villages per sub-county (or sub-location) based on population.

In SA, data were collected through multi-staged sampling methods. In the first stage, we chose three countries and, in the second stage, three districts in Bangladesh (Bagerhat, Jhalokhati, and Satkhira), one district in Nepal (Rupandehi), and one district (Vaishali) in the state of Bihar, India. In total, 38 villages were selected for the study: 14 from Bangladesh, 12 from Bihar (India), and 12 from Nepal. The distribution of sample households in five selected countries of SA and EA is provided in Supplementary Material 5. In all locations, data were collected through a field survey using a comprehensive questionnaire that covered household socioeconomics and farm-level characteristics, climate risks faced, and adaptation strategies employed.

As for all socioeconomic surveys or data collection from family or community representatives, the Institutional Research Ethics Committee (IREC) of the International Maize and Wheat Improvement Center (CIMMYT) classified it as low risk and approved the study and relevant IREC guidelines and regulations were followed. The front page of each questionnaire carried a section where the person interviewed indicated her/his informed consent for the interview. Interviewers were trained and required to read aloud the consent statement to each interviewee before the interview could advance. Participants were informed that they were under no obligation to answer any questions or could stop the interview at any time, without giving any reasons, and ask that any partial data recorded be removed from the records.

### Empirical framework and estimation issues

#### Empirical framework

Farmers in all study locations have applied multiple adaptation strategies; hence, a multivariate probit model is best suited to evaluate the factor influencing the choice of multiple adaptation strategies. Use of univariate logit and probit methodologies for each type of adaptation strategy is not recommended, as it can lead to biased estimates, primarily due to the likelihood of interdependence and simultaneity of adaptation choices^64^. Since more than one adaptation strategy can be applied concurrently, it is likely that the decision to adopt one strategy may influence induce the choice of other strategies. Using a multivariate probit model, this study recognizes the interdependence between choices and the possible association among unobserved random error terms across these equations, while also generating unbiased and efficient estimates^64^. It also reports possible complementarities (positive correlation) and substitutabilities (negative correlation) between various adaptation strategies.

In the study locations, farm households commonly applied five (in some cases, six or seven) major types of adaptation strategies; i.e., changing farming practices (*Y*_1_), use of savings and borrowings (*Y*_2_), reduction in household consumption (*Y*_3_), seek off-farm or other employment options (*Y*_4_), take assistance from government (*Y*_5_), take assistances from non-governmental organizations (*Y*_6_) and sustainable land management (*Y*_7_) only in Kenya. Considering that *i*th farm household (*i* = 1, 2, …, N) has to make a decision on whether to embrace *j*th adaptation strategies (*j* = 1, 2, …, 7), we estimate the following multivariate probit model:

1 $$Y_{ij} = X_{ij}^{^{\prime}} \beta_{j} + \varepsilon_{ij}$$

In Eq. (1), $X_{ij}^{^{\prime}}$ is a 1 × k vector of explanatory variables, $\beta_{j}$ is a k × 1vector of unknown parameters to be estimated, and $\varepsilon_{ij}$ unobserved error terms. In this model specification, the error terms jointly follow a multivariate normal distribution (MVN) with zero conditional mean and variance normalized to unity; i.e., $u_{1} ,\,u_{2} ,\,u_{3} ,\,u_{4} ,\,u_{5} ,u_{6} \,\mathop{\longrightarrow}\limits^{MVN}\left( {0,\,\omega } \right)$. The resulting variance–covariance matrix $\left( \omega \right)$ is:

2 $$\omega = \left[ {\begin{array}{*{20}c} 1 & {\rho_{12} } & {\rho_{13} } & {\rho_{14} } & {\rho_{15} } & {\rho_{16} } \\ {\rho_{21} } & 1 & {\rho_{23} } & {\rho_{24} } & {\rho_{25} } & {\rho_{26} } \\ {\rho_{31} } & {\rho_{32} } & 1 & {\rho_{34} } & {\rho_{35} } & {\rho_{36} } \\ {\rho_{41} } & {\rho_{42} } & {\rho_{43} } & 1 & {\rho_{45} } & {\rho_{46} } \\ {\rho_{51} } & {\rho_{52} } & {\rho_{53} } & {\rho_{54} } & 1 & {\rho_{56} } \\ {\rho_{61} } & {\rho_{62} } & {\rho_{63} } & {\rho_{64} } & {\rho_{65} } & 1 \\ \end{array} } \right]$$

where $\rho$ denotes the pairwise correlation coefficient of the error terms corresponding to any two adaptation strategies. Non-zero correlations in the off-diagonal elements in the covariance matrix justify the application of a multivariate probit instead of a univariate probit for each adaptation strategy.

### Estimation issues: potential endogeneity and multicollinearity

Potential endogeneity and multicollinearity can be crucial issues while estimating the empirical model. In econometric analysis, endogeneity can occur in three possible ways: (1) an omitted variable, (2) measurement errors, and (3) simultaneity^65^. The distinctions among these three possibilities are not easily observed^66^. In our case, it is possible if an explanatory variable may be jointly determined with the decision to adopt an adaptation strategy in the model^67^. As endogeneity results in the inconsistent estimator, an examination of possible endogeneity is required^65,68^. To check for potential endogeneity, we first used the approach suggested in a 1999 study^69^. We specified the potential endogenous variables in our adaptation equation as a function of all other explanatory variables in the model, including a set of instrumental variable, if any, as the following:

3 $$E_{ij} = \gamma \,X_{ij} + \psi \,I_{ij} + \eta_{ij}$$

In Eq. (3), *E* refers to a potential endogenous variable, *X* is the vector of explanatory variables, same as in Eq. (1), and *I* is an instrumental variable that is correlated with the endogenous variable but not with error terms in Eq. (1). After estimating Eq. (3), we calculated the residual term $(\hat{R}_{ij} = \hat{E}_{ij} - E_{ij} )$ from the first stage regression of the endogenous variable and included the residual term as an additional explanatory variable in the model as follows:

4 $$y* = x^{\prime}\beta + \psi \,I_{i} \, + \hat{R}_{ij} \, + \varepsilon$$

The estimates obtained from Eq. (4) are consistent^65^. However, in empirical estimation, it is not possible to get a good instrumental variable. In such a case, we just tested for endogeneity using the only residual term as an additional explanatory variable as suggested by Gujarati and Porter (2009)^70^. In our case, distance from farm household to the agricultural extension service can be taken as an instrument, assuming that it affects the access to information about possible adaptation strategies to farmers, but it will not affect their selection of individual adaptation measures. The empirical results of testing endogeneity are presented in Supplementary Material 6. Given that the residual terms (variable ‘extension_resid’ in Supplementary Material are not significant in all the models, the results confirm no endogeneity. Hence, we continue with the presentation of the analysis without residuals.

Another crucial issue in the estimation of econometric models with multiple variables is possible multicollinearity between explanatory variables^70^. Given that several factors influence the decision to apply the climate change adaptation measures, we need to estimate the multivariate probit models with many explanatory variables. To test the potential multicollinearity among explanatory variables in the model, we applied a condition index^71,72^. As the value of condition index is less than 30, it indicates no serious problem of multicollinearity among the explanatory variables used in our analysis.

## Supplementary Information

Supplementary Information.
