# Supplementary material for: Climate risks and adaptation strategies of farmers in East Africa and South Asia
Source: Sci Rep. 2021 May 18;11:10489. doi: 10.1038/s41598-021-89391-1 (PMC8131377; doi:10.1038/s41598-021-89391-1)
Supplement: Supplementary file 1 — Supplementary Information. [file 41598_2021_89391_MOESM1_ESM.docx]

**SUPPLEMENTARY MATERIAL**

**Supplementary Material 1: Climate risks review in countries under study**

### *Ethiopia*

Droughts and floods are two major climate risks in Ethiopia. During 1900-2010, Ethiopia suffered 12 extreme droughts, which directly implicated in the deaths of almost 4 million people and adversely affected more than 54 million^1^. In the last three decades, there were eight major droughts in Ethiopia, resulting in severe famines ^1-3^ and substantially hampering economic development ^4,5^. The drought of 2015-16 caused massive crop and livestock losses. Floods are another major climatic risk in Ethiopia, but with less severe impacts than droughts^6^. The country has experienced 47 major floods since 1900, killing nearly 2,000 people and severely affecting the lives of more than 2.2 million^1^. Many major floods occurred after the 1980s^7^. Erosion from extreme rainfall causes the loss of an estimated 1.5 billion tons of fertile soil annually, reducing agricultural production substantially^8^. It is estimated that climate change is likely to reduce Ethiopia’s GDP by almost 10%^9^.

### *Kenya*

As in other countries in EA, droughts and floods are the major climate risks in Kenya, with droughts by far the most damaging^2,10^. During 1964 to 2004, Kenya was hit by 11 major droughts that severely affected the livelihoods of more than 1.5 million people and 17 floods affecting about 70,000 people^11,12^. Droughts and floods have become more frequent after 1990^10^, with the former occurring in 1991-92, 1992-1993, 1995-96, 1998-2000, 2004-05, and 2008-09^13,14^. Climatic variability and unpredictable rainfall have negatively affected the country’s nearly completely rainfed agriculture and are expected to worsen^14,15^. Due to El Niño rains, Kenya also suffered heavy floods in 1997-98^13^

### *Bangladesh*

Characterized by flat, low-lying topography and large floodplains, Bangladesh is highly vulnerable to climate risks^16^ such as extreme rainfall and floods, salinity, cyclones, crop pests, and diseases, all of which have severely affected agriculture and the livelihoods of millions of farmers. Severe flooding has become more frequent, causing serious damage to lives, crops, infrastructure, and property^17,18^. Floods, droughts, and high temperatures are anticipated to become more frequent and extreme and may reduce crop yields by as much as 30%, increasing the risk of hunger (<https://ccafs.cgiar.org/bangladesh#.WwTtsu6FPIU>), accessed on 22 September 2019). A 1-m rise in sea level would submerge nearly 18% of the country^19^. Croplands of some 6 million people are exposed to severe salinity, a problem projected to affect 13.6 million by 2050 and 14.8 million by 2080 ^19^. Flooding of riverain areas and sea-level rise will affect large portions of farm land^20,21^. The 2007 floods (<https://www.unicef.org/media/media_40946.html>) that affected India, Bangladesh, Nepal, Bhutan, and Pakistan, provide a glaring example.

As in other South Asian countries, the frequency and severity of floods have been increasing in Bangladesh^18^, which experienced five large floods between 1987 and 2007. The devastating 2007 flood affected almost 20 million people in Bangladesh and caused estimated economic losses of US $1.06 billion^22^. Storm surges often cause devastating floods in the South and southeastern coasts of Bangladesh.

Cyclones are the most destructive climate-induced risk in Bangladesh; more than 52 struck the country from 1960 to 2010^23^ and the worst - the 1970 Bhola cyclone - claimed nearly 0.5 million lives ^24^ and the April 1991 cyclone killed almost 150,000 people. Their frequency has increased recently^25^, including Cyclone Sidr in November 2007, Nargis in May 2008, Aila in May 2009, Mahasen in May 2013, Roanu in May 2016, Mora in May 2017, Fani in May 2019, and Amphan in May 2020 ^24,26^. Sidr affected almost 8.7 million people and claimed approximately 3,300 lives. Such storms occur chiefly in the pre-monsoon months of April and May and the post-monsoon months of October and November^23,24^.

### *India*

Flooding is a major climate risk during the monsoon months in India as in other SA countries ^18^, with northeastern India being most vulnerable and particularly the states in the Ganges Basin, such as Bihar, Uttar Pradesh, and West Bengal. Bihar experiences severe flooding from the Koshi River during the monsoon period. In August 2008, flooding of several villages in Bihar displaced more than three million people^27^. The flood of July 2020 severely impacted the lower Indo-Gangetic-Brahmaputra Plains ^28^, covering large parts of Bihar (6.29%) and Assam (8.99%) in India and also Bangladesh (9.09%) (Lal et al. 2020) and affecting more than 10 million people.

Drought is a major climate risk in India ^29-31^. Almost 20% of India’s total land area is drought-prone^32^, which severely reduces rice production. In July 2002, average rainfall was 49% below the long-term average, causing a drought that affected more than 300 million people across India, particularly in major rice producing states ^32,33^. Large portions of the Indo-Gangetic Plains suffer from both drought and excess water stress. For example, with its flat topography and about 80% of its total annual rainfall falling between July to September, northern Bihar is extremely flood-prone, whereas southern Bihar, parts of which receive less than 1,000 mm annual rainfall, is extremely drought-prone ^34,35,36^. Monsoon season “flash” droughts also have devastating impacts on agriculture^37^. In a study using datasets between 1982 and 2012 from India, Nath et al. (2017) found that the humid subtropical Upper Middle Gangetic Plains region is highly drought-prone (occurrence frequency is 40 to 45%). Agricultural production in this region has suffered a lot due to drought, and it is estimated that at least 50% of the crop losses in Upper Middle Gangetic Plains are caused by drought^38^.

### *Nepal*

Intensive rainfall during July and August usually causes floods in Nepal, particularly in the Terai Region^39^. Historical data show an increase in the scale, intensity, and duration of floods in Nepal^22^. Several past floods – for example, floods in 1985, 1993, and 2004 – had devastating impacts on agriculture and infrastructure and caused severe food insecurity. In July 1993, major flooding in central Nepal which triggered landslides and flooding on the Terai plains^18^. The 1993 flood across Nepal, 2002 flood in central Nepal, 2008 floods in *Koshi* river and in Western Nepal, 2012 flood in *Seti* river, and 2017 flood across Nepal are among the major past floods in Nepal^40^. These floods have adversely affected multiple sectors in Nepal. For instance, the 2008 flood in *Koshi* river affected almost 65,000 people in the eastern region^41^, and the damage from the 2017 flood was estimated to cost approximately US$188 million^42^.

Droughts often affect agriculture and livestock production in Nepal, and data from 1981 to 2012^43^ showed that the frequency and severity of droughts have increased, with the worst and most widespread ones occurring in the summer seasons of 2004, 2005, 2006, 2009, and the winters of 2006, 2008, and 2009. Winter droughts are also common in the Terai ^44^, and the western region of Nepal experienced severe droughts during the winter of 2008-09, reducing wheat and barley production by 50% below the previous year’s crops^45^. Droughts often cause yield variability in crops in Nepal^46,47^.

1 You, G. J.-Y. & Ringler, C. Hydro-economic modeling of climate change impacts in Ethiopia. (International Food Policy Research Institute (IFPRI), 2010).

2 Gebrechorkos, S. H., Hülsmann, S. & Bernhofer, C. Analysis of climate variability and droughts in East Africa using high-resolution climate data products. *Global and Planetary Change* **186**, 103130, doi:<https://doi.org/10.1016/j.gloplacha.2020.103130> (2020).

3 Gebrehiwot, T., van der Veen, A. & Maathuis, B. Spatial and temporal assessment of drought in the Northern highlands of Ethiopia. *International Journal of Applied Earth Observation and Geoinformation* **13**, 309-321, doi:<https://doi.org/10.1016/j.jag.2010.12.002> (2011).

4 Mersha, A. A. & van Laerhoven, F. The interplay between planned and autonomous adaptation in response to climate change: Insights from rural Ethiopia. *World Development* **107**, 87-97, doi:<https://doi.org/10.1016/j.worlddev.2018.03.001> (2018).

5 Seleshi, Y. & Zanke, U. Recent changes in rainfall and rainy days in Ethiopia. *International Journal of Climatology* **24**, 973-983, doi:10.1002/joc.1052 (2004).

6 Weldegebriel, Z. B. & Amphune, B. E. Livelihood resilience in the face of recurring floods: an empirical evidence from Northwest Ethiopia. *Geoenvironmental Disasters* **4**, 1-19 (2017).

7 World Bank. Economics of Adaptation to Climate-Synthesis Report. (The World Bank Group., Washington DC, USA, 2010).

8 Tamene, L. & Vlek, P. L. G. in *Land Use and Soil Resources* (eds Ademola K. Braimoh & Paul L. G. Vlek) 73-100 (Springer Netherlands, 2008).

9 Mideksa, T. K. Economic and distributional impacts of climate change: The case of Ethiopia. *Global Environmental Change* **20**, 278-286, doi:<https://doi.org/10.1016/j.gloenvcha.2009.11.007> (2010).

10 Linke, A. M., Witmer, F. D. & O’Loughlin, J. Do people accurately report droughts? Comparison of instrument-measured and national survey data in Kenya. *Climatic Change* **162**, 1143-1160 (2020).

11 Parry, J.-E., Echeverria, D., Dekens, J. & Maitima, J. Climate risks, vulnerability and governance in Kenya: A review. *Commissioned by: climate risk management technical assistance support project (CRM TASP), joint initiative of bureau for crisis prevention and recovery and bureau for development policy of UNDP* (2012).

12 The Earth Institute. *Kenya natural disaster profile. Retrieved from* [*http://www.ldeo.columbia.edu/chrr/research/profiles/pdfs/kenya_profile1.pdf*](http://www.ldeo.columbia.edu/chrr/research/profiles/pdfs/kenya_profile1.pdf)*. USA* (The Earth Institute, University of Columbia, USA, n.a.).

13 Orindi, V. A. & Ochieng, A. Case Study 5: Kenya Seed Fairs as a Drought Recovery Strategy in Kenya. *IDS Bulletin* **36** (2005).

14 Ochieng, J., Kirimi, L. & Mathenge, M. Effects of climate variability and change on agricultural production: The case of small scale farmers in Kenya. *NJAS - Wageningen Journal of Life Sciences* **77**, 71-78, doi:<https://doi.org/10.1016/j.njas.2016.03.005> (2016).

15 Ochieng, J., Kirimi, L. & Makau, J. Adapting to climate variability and change in rural Kenya: farmer perceptions, strategies and climate trends. *Natural Resources Forum* **41**, 195-208, doi:10.1111/1477-8947.12111 (2017).

16 Brammer, H. Floods in Bangladesh: geographical background to the 1987 and 1988 floods. *Geographical journal*, 12-22 (1990).

17 Khalequzzaman, M. Recent floods in Bangladesh: Possible causes and solutions. *Natural Hazards* **9**, 65-80 (1994).

18 Mirza, M. M. Q. Climate change, flooding in South Asia and implications. *Regional environmental change* **11**, 95-107 (2011).

19 Khan, I. A., Ali, Z., Asaduzzaman, M. & Bhuyan, M. H. R. The social dimensions of adaptation to climate change in Bangladesh. Development and climate change. (World Bank, Washingtion DC, USA, 2010).

20 Ruane, A. C. *et al.* Multi-factor impact analysis of agricultural production in Bangladesh with climate change. *Global Environmental Change* **23**, 338-350 (2013).

21 Thomas, T. S. *et al.* *Agriculture and adaptation in Bangladesh: Current and projected impacts of climate change*. Vol. 1281 (Intl Food Policy Res Inst, 2013).

22 Dewan, T. H. Societal impacts and vulnerability to floods in Bangladesh and Nepal. *Weather and Climate Extremes* **7**, 36-42, doi:<https://doi.org/10.1016/j.wace.2014.11.001> (2015).

23 Farukh, M., Hossen, M. & Ahmed, S. Impact of extreme cyclone events on coastal agriculture in Bangladesh. *Progressive Agriculture* **30**, 33-41 (2019).

24 Hossain, I. & Mullick, A. R. Cyclone and Bangladesh: A Historical and Environmental Overview from 1582 to 2020. **25** (2020).

25 Ali, A. Vulnerability of bangladesh to climate change and sea level rise through tropical cyclones and storm surges. *Water, Air, and Soil Pollution* **92**, 171-179 (1996).

26 United Nations. Cyclone Sidr Bangladesh situation, Report No. 12 (Office for the Coordination of the Humanitarian Affairs (OCHA), Bangkok, Thailand, 2007).

27 Action Aid. Bihar floods 2008 needs assessment report. <http://www.actionaidusa.org/assets/pdfs/bihar_floods_needs_assessment.pdf>. (Action Aid, India, Bangalore, 2008).

28 Lal, P., Prakash, A. & Kumar, A. Google Earth Engine for concurrent flood monitoring in the lower basin of Indo-Gangetic-Brahmaputra plains. *Natural Hazards* **104**, 1947-1952 (2020).

29 Mishra, V. *et al.* Drought and famine in India, 1870–2016. *Geophysical Research Letters* **46**, 2075-2083 (2019).

30 Thomas, J. & Prasannakumar, V. Temporal analysis of rainfall (1871–2012) and drought characteristics over a tropical monsoon-dominated State (Kerala) of India. *Journal of Hydrology* **534**, 266-280 (2016).

31 Zhang, X., Obringer, R., Wei, C., Chen, N. & Niyogi, D. Droughts in India from 1981 to 2013 and implications to wheat production. *Scientific reports* **7**, 1-12 (2017).

32 Ward, P. S., Ortega, D. L., Spielman, D. J. & Singh, V. Heterogeneous Demand for Drought-Tolerant Rice: Evidence from Bihar, India. *World Development* **64**, 125-139, doi:<https://doi.org/10.1016/j.worlddev.2014.05.017> (2014).

33 Indian Meteorological Department. Southwest Monsoon 2002 End of season report. (2002).

34 Government of Bihar. State Action Plan on Climate Change: Building Resilience through Development. (Government of Bihar, Bihar, India, 2012).

35 Aryal, J. P. *et al.* Adoption of multiple climate-smart agricultural practices in the Gangetic plains of Bihar, India. *International Journal of Climate Change Strategies and Management* **10**, 407-427, doi:10.1108/ijccsm-02-2017-0025 (2018).

36 Sehgal, V. K., Singh, M. R., Chaudhary, A., Jain, N. & Pathak, H. Vulnerability of Agriculture to Climate Change: District Level Assessment in the Indo-Gangetic Plains. (Indian Agricultural Research Institute, Indian Council of Agricultural Research, New Delhi 110 012, 2013).

37 Mahto, S. S. & Mishra, V. Dominance of summer monsoon flash droughts in India. *Environmental Research Letters* **15**, 104061 (2020).

38 Nath, R., Nath, D., Li, Q., Chen, W. & Cui, X. Impact of drought on agriculture in the Indo-Gangetic Plain, India. *Advances in Atmospheric Sciences* **34**, 335-346 (2017).

39 Dingle, E. *et al.* Dynamic flood topographies in the Terai region of Nepal. *Earth Surface Processes and Landforms* **45**, 3092-3102 (2020).

40 Thapa, S., Shrestha, A., Lamichhane, S., Adhikari, R. & Gautam, D. Catchment-scale flood hazard mapping and flood vulnerability analysis of residential buildings: The case of Khando River in eastern Nepal. *Journal of Hydrology: Regional Studies* **30**, 100704, doi:<http://www.sciencedirect.com/science/article/pii/S2214581820301786>. (2020).

41 Government of Nepal. . Nepal Disaster Report 2009. (Kathmandu, Nepal, 2009).

42 Government of Nepal. Nepal Flood 2017: Post Flood Recovery Needs Assessment. (Kathmandu, Nepal, 2017).

43 Dahal, P. *et al.* Drought risk assessment in central Nepal: temporal and spatial analysis. *Natural hazards* **80**, 1913-1932 (2016).

44 Government of Nepal. Agriculture Development Strategy (ADS), 2014. (Ministry of Agricultural Development, Singa Darbar, Kathmandu, Nepal, 2014).

45 Wang, S.-Y., Yoon, J.-H., Gillies, R. R. & Cho, C. What caused the winter drought in western Nepal during recent years? *Journal of Climate* **26**, 8241-8256 (2013).

46 Hamal, K. *et al.* Assessment of drought impacts on crop yields across Nepal during 1987–2017. *Meteorological Applications* **27**, e1950 (2020).

47 Khatiwada, K. R. & Pandey, V. P. Characterization of hydro-meteorological drought in Nepal Himalaya: A case of Karnali River Basin. *Weather and Climate Extremes* **26**, 100239, doi:<https://doi.org/10.1016/j.wace.2019.100239> (2019).

**Supplementary Material 2: Interdependence among risk coping strategies**

**Supplementary Material 2 Table 1: Interdependence among risk coping strategies**

| Coping strategies | Bangladesh | India | Nepal | Ethiopia | Kenya |
| --- | --- | --- | --- | --- | --- |
| ‘Change in farming practices’ and ‘Use past savings or borrowing money’ | -0.167**  (0.081) | 0.249***  (0.072) | 0.233***  (0.089) | 0.127***  (0.040) | 0.261***  (0.095) |
| ‘Change in farming practices’ and ‘Reduce household consumption’ | 0.150*  (0.086) | -0.161**  (0.079) | 0.217***  (0.078) | 0.314***  (0.049) | -0.090  (0.071) |
| ‘Change in farming practices’ and ‘Seek off-farm or other employment’ | -0.345***  (0.074) | 0.138*  (0.077) | -0.258***  (0.087) | -0.125**  (0.059) | -0.256***  (0.093) |
| ‘Change in farming practices’ and ‘Take assistance from government’ | -0.226**  (0.093) | 0.144  (0.131) | -0.119  (0.122) | -0.063  (0.064) | - |
| ‘Use past savings or borrowing money’ and ‘Reduce consumption’ | 0.176**  (0.081) | -0.176**  (0.084) | -0.207**  (0.085) | -0.131**  (0.063) | -0.456  (0.348) |
| ‘Use past savings or borrowing money’ and ‘Seek off-farm or other employment’ | -0.072  (0.081) | 0.064  (0.079) | -0.145  (0.129) | -0.111  (0.070) | -0.176**  (0.085) |
| ‘Use past savings or borrowing money’ and ‘Take assistance from government’ | 0.131  (0.096) | -0.281***  (0.106) | 0.302**  (0.145) | 0.084  (0.060) | - |
| ‘Reduce household consumption’ and ‘Seek off-farm or other employment’ | 0.239***  (0.084) | 0.181**  (0.077) | 0.167  (0.133) | -0.269***  (0.085) | -0.452*  (0.274) |
| ‘Reduce household consumption’ and ‘Take assistance from government’ | -0.071  (0.114) | -0.103  (0.119) | 0.307**  (0.123) | 0.166**  (0.074) | - |
| ‘Seek off-farm or other employment’ and ‘Take assistance from government’ | 0.278***  (0.089) | 0.019  (0.110) | 0.096  (0.146) | 0.082  (0.073) | - |
| ‘Change in farming practices’ and ‘Take assistance from NGOs’ | -0.043  (0.091) | - | - | - | - |
| ‘Use past savings or borrowing money’ and ‘Take assistance from NGOs’ | -0.032  (0.101) | - | - | - | - |
| ‘Reduce household consumption’ and ‘Take assistance from NGOs’ | 0.067  (0.096) | - | - | - | - |
| ‘Seek off-farm or other employment’ ‘Take assistance from NGOs’ | 0.219**  (0.098) | - | - | - | - |
| ‘Take assistance from government’ and ‘Take assistance from NGOs’ | 0.635***  (0.071) | - | - | - | - |
| ‘Change in farming practices’ and ‘Sustainable land management’ | - | - | - | - | 0.192**  (0.090) |
| ‘Use past savings or borrowing money’ and ‘Sustainable land management’ | - | - | - | - | 0.159  (0.112) |
| ‘Reduce household consumption’ and ‘Sustainable land management’ | - | - | - | - | 0.040  (0.091) |
| ‘Seek off-farm or other employment’ and ‘Sustainable land management’ | - | - | - | - | 0.171**  (0.083) |

Note:

1. For Bangladesh: Likelihood ratio test of rho21 = rho31 = rho41 = rho51 = rho61 = rho32 = rho42 = rho52 = rho62 = rho43 = rho53 = rho63 = rho54 = rho64 = rho65 = 0; chi2(15) = 188.24; Prob > chi2 = 0.0000
2. For Bihar (India): Likelihood ratio test of rho21 = rho31 = rho41 = rho51 = rho32 = rho42 = rho52 = rho43 = rho53 = rho54 = 0; chi2(10) = 148.51; Prob > chi2 = 0.0000
3. For Nepal: Likelihood ratio test of rho21 = rho31 = rho41 = rho51 = rho32 = rho42 = rho52 = rho43 = rho53 = rho54 = 0; chi2(10) = 108.23; Prob > chi2 = 0.0000
4. For Ethiopia: Likelihood ratio test of rho21 = rho31 = rho41 = rho51 = rho32 = rho42 = rho52 = rho43 = rho53 = rho54 = 0; chi2(10) = 99.11; Prob > chi2 = 0.0000
5. For Kenya: Likelihood ratio test of rho21 = rho31 = rho41 = rho51 = rho32 = rho42 = rho52 = rho43 = rho53 = rho54 = 0; chi2(10) = 83.20; Prob > chi2 = 0.0000
6. *, **, and *** refer to 10, 5 and 1% level of significance and standard error in parenthesis

**Supplementary Material 3: Descriptive statistics**

**Supplementary Material 3 Table 1: Explanatory variables used in the MVP model**

| **Variables** | **Bangladesh** | **India** | **Nepal** | **Ethiopia** | **Kenya** | **Variable descriptions** |
| --- | --- | --- | --- | --- | --- | --- |
| MHH (D) | 0.89  (0.31) | 0.91  (0.29) | 0.78  (0.41) | 0.92  (0.26) | 0.81  (0.39) | 1 if male-headed households, 0 otherwise |
| Age (C) | 46.93  (13.30) | 50.51  (13.63) | 50.19  (13.69) | 44.92  (12.90) | 52.69  (14.28) | Age of HH head in years |
| Agri occup (D) | 0.68  (0.37) | 0.91  (0.22) | 0.76  (0.39) | 0.93  (0.25) | 0.72  (0.45) | 1 if agriculture is main occupation, 0 otherwise |
| Education |  |  |  |  |  |  |
| Illiterate (D) | 0.29  (0.45) | 0.38  (0.49) | 0.48  (0.50) | 0.41  (0.48) | 0.07  (0.25) | Education level of the HH head Illiterate |
| Primary (D) | 0.33  (0.47) | 0.09  (0.29) | 0.19  (0.38) | 0.34  (0.47) | 0.17  (0.38) | Up to primary education (Grade 1-5) |
| Secondary (D) | 0.31  (0.46) | 0.38  (0.48) | 0.28  (0.45) | 0.21  (0.41) | 0.49  (0.50) | Up to secondary education (Grade 6-10) |
| Higher (D) | 0.07  (0.26) | 0.15  (0.36) | 0.05  (0.22) | 0.04 (0.19) | 0.25  (0.44) | Higher secondary or above (Grade 11 and above) |
| Labor (C) | 3.27  (1.27) | 6.4  (2.88) | 4.5 (2.19) | 5.63  (2.17) | 4.94  (2.33) | HH labor availability (adult equivalents) |
| Land (C) | 0.44  (0.48) | 0.51  (0.52) | 0.49  (0.78) | 1.92  (1.97) | 1.03  (1.24) | Total farm land operated (ha) |
| Livestock (C) | 0.92  (1.19) | 0.7  (0.82) | 1.25  (1.56) | 4.58  (4.57) | 1.55  (1.75) | Livestock owned (tropical livestock units, TLU) |
| Assets (C) | 0.43  (0.75) | 0.3  (0.53) | 0.45  (0.68) | 0.29  (0.63) | 0.28  (0.67) | Household asset index |
| Food secure (D) | 0.72  (0.44) | 0.74  (0.31) | 0.89  (0.30) | 0.58  (0.49) | 0.45  (0.50) | 1 if the household is food secure, 0 otherwise |
| Training (D) | 0.07  (0.44) | 0.24  (0.31) | 0.04 (0.30) | 0.93 (0.49) | 0.76 (0.50) | 1 if participated in training, 0 otherwise |
| Credit (D) | 0.69  (0.46) | 0.39 (0.49) | 0.44 (0.49) | 0.45 (0.49) | 0.32 (0.47) | 1 if access to credit facility, 0 otherwise |
| Member (D) | 0.39  (0.49) | 0.11 (0.31) | 0.46 (0.51) | 0.61 (0.44) | 0.72 (0.41) | 1 if member in farmers organizations, 0 otherwise |
| Market*(C) | 3.54  (4.02) | 2.46 (1.49) | 5.12 (4.09) | 93.17 (84.77) | 73.59 (52.12) | Distance to nearest main market from house (km) |
| Extension* (C) | 9.16  (8.79) | 4.67 (3.71) | 7.56 (8.61) | 30.24 (31.34) | 65.54 (56.37) | Distance to agricultural extension service (km) |

1. C and D refer to continuous and dummy variables, respectively. * For Ethiopia and Kenya, distance to market and extension service is measured in walking minutes.
2. To capture the effect of wealth on the choice of risk coping strategies, we constructed household asset index using principal component analysis (for detail, see <https://www.stata.com/manuals13/mvpca.pdf>). We included most of the household assets such as tractors, cars, television, water pump, motorbike, etc., for constructing household asset index.
3. Adult equivalent
4. Tropical livestock unit (TLU): calculated using Chilonda, P., Otte, J., 2006. Indicators to monitor trends in livestock production at national, regional, and international levels. Livestock research for Rural Development 18. Article number 117. Accessed at <http://www.lrrd.org/lrrd18/8/chil18117.htm>

The descriptive statistics and description of the explanatory variables used in the study is shown in the table above. Substantial differences are observed in the gender of the headship among the farm households in these five countries in SA and EA. ThepercentageofFHHsis much higher in Nepal (22%) and Kenya (19%) compared to India (9%)and Bangladesh and Ethiopia (8% each). FewerFHHsin India and Bangladesh compared to Nepal can be due to more conservative socio-cultural norms in these countries([Mallick and Rafi, 2010](#_ENREF_31); [Aryal *et al.*, 2014](#_ENREF_5)), and also due to the out-migration of a large number of male members from Nepal ([Lama *et al.*, 2017](#_ENREF_26)). In Bangladesh, the practice of ‘*Purdah*’ confines women within the homestead as they mostly require men’s permission for most of the works([Mahmud *et al.*, 2012](#_ENREF_29)). In EA, FHHs are increasing due to male out-migration to seek employment, widowhood, divorce, and other family disruptions([FAO, 2011](#_ENREF_16); [Kassie *et al.*, 2014](#_ENREF_23)).

The average age of the household head is relatively similar (45 to 47 years) for SA, while it is 45 for Ethiopia and 53 for Kenya. Literacy plays an important role in the adoption of technology([Aryal *et al.*, 2018b](#_ENREF_8); [Aryal *et al.*, 2018c](#_ENREF_9))and climate adaptation([Mulwa *et al.*, 2017](#_ENREF_35)). Around 38% of the household head is illiterate in Bihar, 29% in Bangladesh, and 48% in Nepal. Low level of illiteracy level in Nepal may be due to a larger percentageofhouseholdsareFHHsin the country and females have a lower level of literacy. In EA, we found that 44% of the household head are illiterate in Ethiopia, while it is only 7% in Kenya. The average landholding ranges between 0.44 and 0.51 hectaresinSA. In EA, average landholding is slightly higher: 1.92 hectares in Ethiopia and 1.03 hectares in Kenya. Average livestock asset measured in terms of tropical livestock unit (TLU) is highest for Nepal (1.25), followed by Bangladesh (0.92) and Bihar (0.70). In EA, the TLU is much higher in Ethiopia (4.58) than in Kenya (1.55). The household assets index is relatively similar across these countries- Nepal (0.45), Bangladesh (0.43), Bihar (0.30), Ethiopia (0.29), and Kenya (0.28).

Food security across three SA countries is similar: slightly higher for Nepal (79%), followed by Bihar (74%) and Bangladesh (72%). However, it is much lower in Ethiopia (58%) and Kenya (45%). The number of households who participated in climate change-related agricultural training in SAis extremely low, particularly for Nepal (4%) and Bangladesh (7%); at least for Bihar, the households who participated in such training was better (24%). In EA, it is much higher (93% in Ethiopia and 76% in Kenya) because of the intervention of projects on the scaling of conservation agriculture technology.

Access to credit helps in overcoming the liquidity constraint and thus facilitates climate adaptation. In Bangladesh, about 69% of the household had access to credit, while it was 39% in Bihar, 44% in Nepal, 45% in Ethiopia, and 32% in Kenya. Access to credit in Bangladesh is high because Bangladesh is a [pioneer](https://www.google.com/search?rlz=1C1GCEA_enMX875MX875&sxsrf=ACYBGNQyb6QmSAF7Qpdp_CQBTRNTPCMj_g:1576587525459&q=pioneer&spell=1&sa=X&ved=2ahUKEwij2qWb3rzmAhXUZCsKHVnpAycQkeECKAB6BAgTECU) in microcredit. Membership in institutions plays an important role in networking and sharing information on agricultural practices. In EA (61% in Ethiopia and 72% in Kenya), more farmers are members of farmers organizations compared to SA(Nepal-46%; Bangladesh-39% and Bihar-11%). Access to market and agricultural extension services significantly influences the adoption of agricultural technology and climate adaptation strategies. The result shows that the distance to market (in kilometers) is shorter than the distance to agricultural extension services in SA. The distances to market and extension services are 5.12 and 8.61 km in Nepal, 3.54 km and 9.16 km in Bangladesh, and 2.46 km and 4.67 km in Bihar, respectively. In EA, distance to market (in walking distance in minutes) is longer than the distance to agricultural extension services. The distance to market and extension services is 93.17 and 30.24 minutes in Ethiopia, 73.59and 65.54minutesin Kenya, respectively.

**Supplementary Material 4**

**Macro Indicators for climate risk, vulnerability and adaptive capacity**

**Supplementary Material 4 Figure 1: Macro risk and resilance indicators**


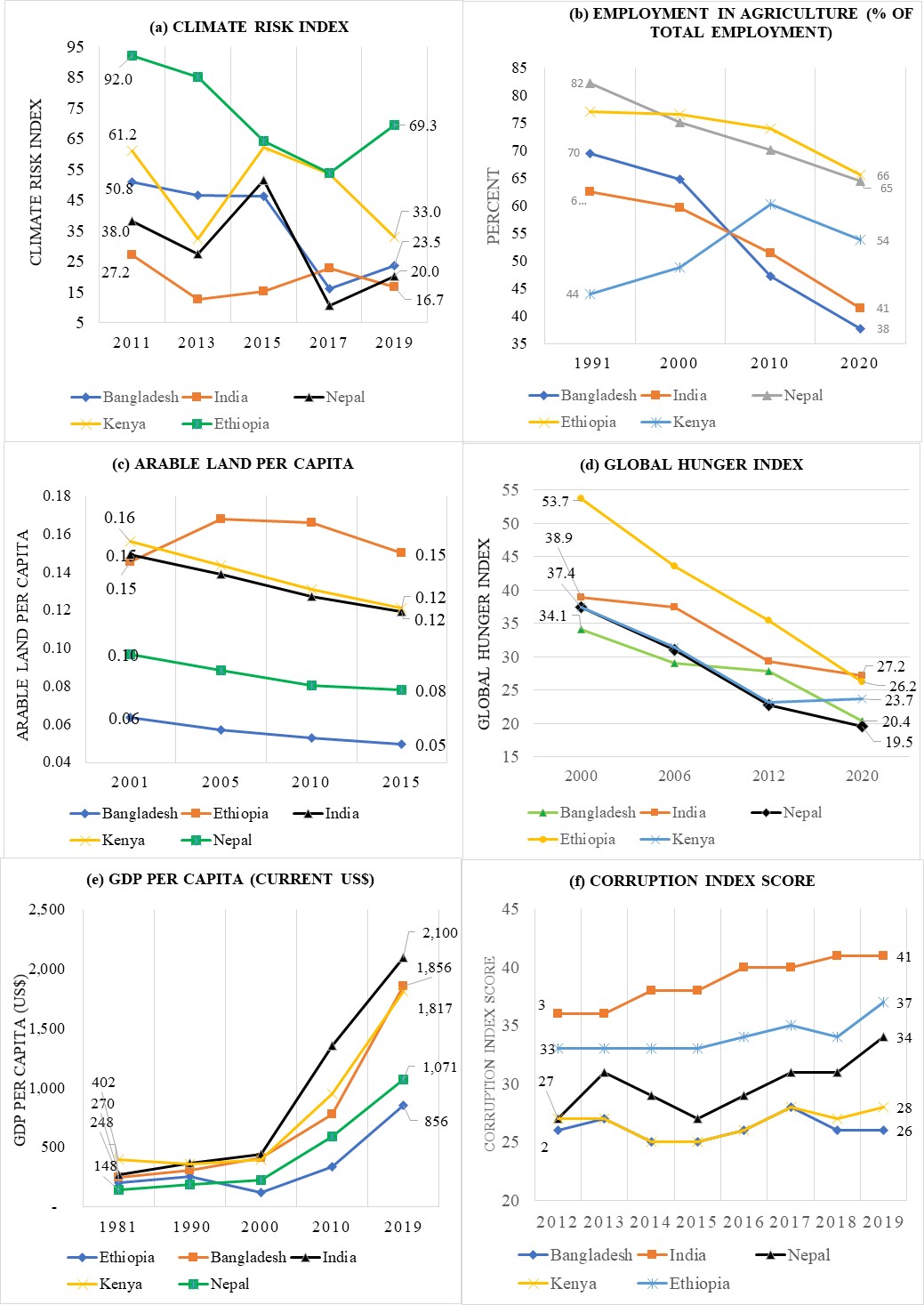


Source: World Development Indicator, Germanwatch and Global Risk Profile

In 2019 the climate risk score index was 69.3 for Ethiopia, followed by Kenya (33.0), Bangladesh (23.5), Nepal (20), and India (16.7), which is relatively high compared to other regions of the world. The climate risk score index displays a fluctuation in climate risk from year to year for all the countries under study. Ethiopia has the highest level of exposure, followed by Kenya, which explains the high level of vulnerability to climate risk among households in eastern Africa. As agriculture is most vulnerable to climate risk, the percentage of the population dependent on agriculture for their livelihood indicate the level of exposure to severe climate shocks. The percentage of the population dependent on agriculture has declined sharply during the last three decades except in Kenya, where it increased from 44% to 54%. In Bangladesh, the percentage of the population in agriculture declined from 70% in 1991 to 38 in 2020. During the same period, the percentage of the population in agriculture declined from 63% to 41% in India, from 82% to 65% in Nepal, and from 77% to 66% in Ethiopia. Despite such dramatic decline, still, a significant portion of the population is dependent on agriculture for their livelihood and, hence, more vulnerable to climate shocks. Farmers in both regions are smallholders, as indicated by the small size of arable land per capita The low per capita arable land explains poor resilience and adaptive capacity of agricultural land and ensures food security, and reduces household vulnerability. The percentage of arable land in Bangladesh and India is as high as 53% and 60%, respectively, while it is 15% for Ethiopia and Nepal and only 10% in Kenya [^46^](#_ENREF_46). The arable land per capita is very small for all the countries under study and shows a decline, for example, in 2015, the per capita arable land is lowest in Bangladesh (0.05 hectare), followed by Nepal (0.08 ha), India, Kenya (0.12 ha) and Ethiopia (0.15). The global hunger index reflects the level of hunger and poverty; therefore, the household in a country with a higher national hunger index has limited or no capacity to adapt.

National capacity to support farmers to adapt climate change matters a lot in reducing farmers’ vulnerability to climate change. The income per capita indicates the capacity of the nation to invest in the adaptive and coping capacity. In south Asia and east Africa, the GDP per capita is very low, although there has been a significant increase. In Ethiopia, the GDP per capita increased from US$ 124 in 2000 to US$ 856, and during the same period, it increased from US $229 to US$ 1,071 in Nepal, and it increased from US$ 397 to US$ 1,817 in Kenya, from US$ 418 to US$ 1,856 in Bangladesh and from US$ 443 to US$ 2100 in India. Despite rapid economic growth, these countries still lack enough resources to invest in improving the households’ resilience capacity. Although neglected in much literature, good governance plays an important role in managing risk and improving the welfare of the people living in the country [^49^](#_ENREF_49)^,^[^50^](#_ENREF_50). Hence we analysed the corruption index (CI) score to understand the good governance situation in the country under study. The CI score is below 50 for all countries, which shows the lack of poor governance in these countries and places them in a vulnerable situation, and limits their capacity to effectively invest in improving the resilience of the country against shocks. Over the last decades, the CI score has improved marginally for India, Nepal, and Ethiopia while it has remained consistently low in Bangladesh and Kenya. As of 2019, the CI score best for India, which stood at 40, followed by Ethiopia (40), Nepal (29), and Kenya and Bangladesh (26). Additionally, we evaluated several other national levels of adaptive and exposure indicators such as share of irrigated land, the share of agriculture to GDP, employment in the agricultural sector, external debt stock to GNI ratio, the reserve to total external debt ratio. These indicators also reflect a higher level of exposure and limited capacity to cope with the climate shock (see Supplementary Material 4 Figure 2)

**Supplementary Material 4 Figure 2- Macro risk and vulnerability**

**
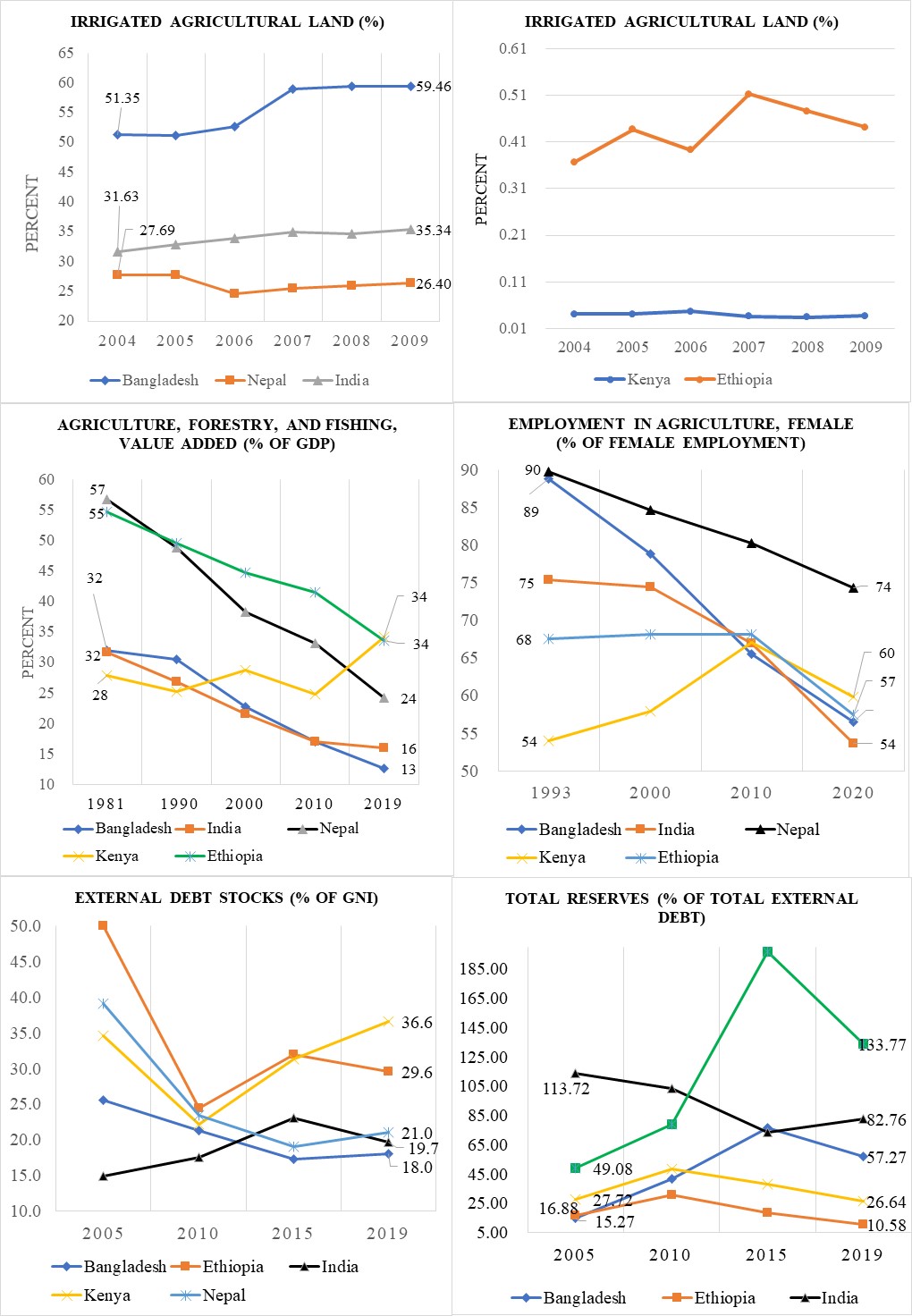
**

*Irrigated land:* Irrigation helps farmers adapt to climate risks such as variability in rainfall, drought, and heat stress; hence, the share of irrigated land defines the country’s resilience. South Asia is slightly better than East Africa in terms of irrigated land. In India, over 59% of the land is irrigated, while it is only 35.3% in Bangladesh and 26.4% in Nepal. However, the situation is worst in east Africa; the percentage of irrigated land is less than 0.5%. There has been no or marginal increase in the irrigated land during the last decades, which is discouraging.

*Contribution of agriculture GDP:* Share of agriculture, forestry, and fishing, value-added to GDP declined sharply in all the country under study except for Kenya, where its share to GDP increased from 28% in 1981 to 34% in 2019. As of 2019, the share of agriculture to GDP stood at 34% for Ethiopia and Kenya and 24% in Nepal, 16% in India, and 13% in Bangladesh.

*Employment of female in the agricultural sector:* The percentage of the female population dependent on agriculture has declined sharply during the last three decades. Despite such dramatic decline, a significant portion of the female population is dependent on agriculture for their livelihood, hence is more vulnerable to climate shocks.

*External debt:* Total external debt stock provides an idea about the dependency on the foreign capital on the one hand and the amount of resources the country has to spend on servicing the debt. As of 2019, the external debt stock to GNI was 36.6% for Ethiopia, 29.6 for Kenya, 21% for Nepal, 19.7% for India, and 18% for Bangladesh. Furthermore, we looked into the ratio between total reserve to debt, which indicates the extent to which the reserve can cover the debt, and the picture is gloomy. Although the reserve to total external debt has increased over the years, it is still low; for example, in reserve to debt ratio is 10.6% for Ethiopia, 26.6 for Kenya, 57.3 for Bangladesh, 82.8 for Bangladesh, and 133.8 for Nepal.

**Supplementary Material 5: Distribution of sample households in five selected countries of South Asia and East Africa**

| **Bangladesh** | | **India (Bihar)** | | **Nepal** | | **Ethiopia** | | **Kenya** | |
| --- | --- | --- | --- | --- | --- | --- | --- | --- | --- |
| **Villages** | **Sample size** | **Villages** | **Sample size** | **Villages** | **Sample size** | **Region** | **Sample size** | **County/District** | **Sample size** |
| Boro Galua | 32 | Bhatha Dasi | 63 | Aahirauli | 50 | Amhara | 329 | Bungoma | 137 |
| Burigoalini | 45 | Bilandpur | 68 | Bairiyan | 50 | Benishangual-Gumuz | 99 | Embu | 93 |
| Chandipur | 66 | Dedhpur | 46 | Bhaglapur | 32 | Oromia | 1359 | Tharaka | 81 |
| Dumuria | 64 | Dhabhaich | 46 | Dewapar | 59 | SNNP | 500 | Meru | 81 |
| Durgapur | 8 | Laxminarayanpur | 44 | Dhakdahi | 92 |  |  | Siaya | 143 |
| Gabgasia | 66 | Mirpur | 55 | Haraiya | 47 |  |  |  |  |
| Gopalpur | 32 | Mukundpur | 69 | Hati Bangai | 33 |  |  |  |  |
| Hatsala | 28 | Panapur Camp | 56 | Mahuwari | 71 |  |  |  |  |
| Horinagor | 45 | Raja Pakar | 70 | Parasi Thuga | 66 |  |  |  |  |
| Jagannathpur | 64 | Rampur Ratnagar | 45 | Razadh | 36 |  |  |  |  |
| Joka | 40 | Rasalpour | 48 | Rehara | 48 |  |  |  |  |
| Sreefal Kathi | 45 | Varishpur | 31 | Samrahana | 47 |  |  |  |  |
| Tarabunia | 45 |  |  |  |  |  |  |  |  |
| Teligati | 50 |  |  |  |  |  |  |  |  |
| Total | 630 |  | 641 |  | 631 |  | 2287 |  | 535 |

**Supplementary Material 6: Pooled model and endogeneity checks**

We estimated the multivariate probit model with pooled data, using country dummies for the study countries. As there are variations regarding the use of adaptation strategies across the study countries, we need to club together some of the climate adaptation strategies together while doing the pooled analysis. For example, of the major climate adaptation strategies adopted by the farm households [i.e., change in farming practices (Y1), use past savings/borrowings (Y2), reduce consumption (Y3), seek off-farm or other employment (Y4), take assistance from government (Y5), take assistance from non-governmental organizations (Y6) and sustainable land management (Y7)], we have no observations for climate adaptation strategies Y5 and Y6 for Kenya. Similarly, we have no observations for Y7 for all study countries except Kenya. Therefore, in the pooled analysis, Y1 in the case of Kenya also includes Y7 also. In addition, we also merged Y5 and Y6 together for all countries. We did this to overcome the problem of no observations in this case.

**Supplementary Material 6 Table 1: Determinants of the choice of adaptation strategies by farm households (pooled analysis)**

|  | Change in farming practices | Use past savings/borrowings | Reduce consumption | Seek employment | Institutional support (Y5+Y6) |
| --- | --- | --- | --- | --- | --- |
| Male-headed HH | 0.228*** | -0.303** | -0.209 | 0.396*** | 0.017*** |
|  | (0.051) | (0.149) | (0.211) | (0.097) | (0.006) |
| Age of HH head | -0.002 | -0.001 | 0.009** | -0.014** | -0.007 |
|  | (0.004) | (0.006) | (0.004) | (0.007) | (0.008) |
| Occup in Agri | 0.302*** | 0.219** | 0.167*** | -0.135 | -0.236 |
|  | (0.077) | (0.106) | (0.051) | (0.186) | (0.234) |
| Edu: Primary | 0.151 | 0.188 | 0.297*** | -0.166 | -0.211 |
|  | (0.139) | (0.190) | (0.098) | (0.215) | (0.313) |
| Edu: Secondary | 0.197*** | 0.250** | 0.201 | 0.302** | -0.299 |
|  | (0.065) | (0.121) | (0.223) | (0.148) | (0.305) |
| Edu: Higher | -0.189*** | 0.224*** | -0.402*** | 0.376*** | 0.123** |
|  | (0.058) | (0.079) | (0.133) | (0.115) | (0.046) |
| Labor | 0.096*** | -0.044 | 0.047 | -0.133*** | 0.059 |
|  | (0.030) | (0.054) | (0.051) | (0.041) | (0.061) |
| Land | 0.118** | -0.301*** | -1.316*** | 0.091 | -0.102 |
|  | (0.051) | (0.095) | (0.350) | (0.082) | (0.113) |
| Livestock | 0.134*** | 0.213*** | -0.095 | -0.102 | 0.077 |
|  | (0.041) | (0.050) | (0.091) | (0.092) | (0.073) |
| Asset | 0.246*** | -0.345*** | -0.318** | 0.161** | 0.093 |
|  | (0.078) | (0.097) | (0.152) | (0.079) | (0.100) |
| Food secure | 0.091*** | -0.102 | -0.220*** | -0.201 | 0.403 |
|  | (0.029) | (0.204) | (0.068) | (0.231) | (0.411) |
| Training | 0.298*** | 0.375 | -0.117* | 0.130** | 0.036** |
|  | (0.085) | (0.417) | (0.062) | (0.057) | (0.016) |
| Credit | -0.144* | 0.225** | 0.163 | -0.301*** | -0.210 |
|  | (0.078) | (0.105) | (0.171) | (0.087) | (0.209) |
| Membership | 0.310*** | 0.296** | -0.165 | 0.166** | 0.201** |
|  | (0.097) | (0.146) | (0.163) | (0.075) | (0.096) |
| Market | 0.046*** | -0.058 | 0.033 | 0.052*** | -0.167*** |
|  | (0.015) | (0.055) | (0.037) | (0.013) | (0.048) |
| Extension | 0.041*** | -0.020 | 0.060 | 0.046** | 0.061** |
|  | (0.013) | (0.019) | (0.058) | (0.021) | (0.030) |
| Nepal dummy^#^ | 0.053*** | -0.031** | -0.076*** | -0.025 | -0.135** |
|  | (0.015) | (0.014) | (0.022) | (0.019) | (0.057) |
| India dummy^#^ | -0.303 | 0.013 | 0.051* | 0.044 | 0.156*** |
|  | (0.275) | (0.044) | (0.027) | (0.051) | (0.039) |
| Kenya dummy^#^ | 0.211*** | -0.068*** | 0.091*** | -0.075*** | -0.315*** |
|  | (0.064) | (0.023) | (0.029) | (0.025) | (0.087) |
| Ethiopia dummy^#^ | -0.158*** | -0.015** | -0.021** | -0.033*** | -0.331*** |
|  | (0.049) | (0.007) | (0.010) | (0.011) | (0.066) |
| Constant | -0.833*** | -1.245*** | -2.102*** | -2.485*** | -0.978*** |
|  | (0.271) | (0.326) | (0.571) | (0.605) | (0.319) |
| No. of observation | 4724 | 4724 | 4724 | 4724 | 4724 |

Log likelihood = -3169.39; Wald chi2(100) = 1260.45; Prob > chi2 = 0.0000

Note:

1. *, **, *** refer to 10, 5, and 1% level of significance, respectively. Standard errors are in parentheses.
2. # base category is Bangladesh.

According to Wooldridge (2002), endogeneity can occur in three possible ways: i) omitted variable, ii) measurement errors, and iii) simultaneity. Deaton (1995) explained that it is not easy to make distinctions among these three possibilities. Though the instrumental variable approach is often discussed in theory as the best method to correct for endogeneity, it is very difficult to find an instrument in practice. Some authors consider education as an endogenous variable. However, we did not check it as we did not have parents’ education in our data. Several research articles published in internationally renowned journals also used education, ignoring its possible endogeneity effect. Moreover, as we have included most of the variable that can influence them, we hope that will control for such effects. Hence, we resorted to use the Rivers and Voung (1988) approach without any particular instrument and applied it for the most possible variable that can cause endogeneity. We followed some of the previous empirical studies, such as Abdulai and Huffman (2014) and Aryal et al. (2018). Therefore, we estimated two probit models for participation in training and food security status separately. Then, we calculated the estimated residual terms for both of these variables. We used these two residual terms as additional variables in the model and check whether the residual terms are significant or not. If the residual terms are not significant, it confirms that there is no serious endogeneity.

**Supplementary Material 6 Table 2: Determinants of the choice of adaptation strategies by farm households (pooled analysis with endogeneity corrections)**

|  | Change in farming practices | Use past savings/borrowings | Reduce consumption | Seek employment | Institutional support (Y5+Y6) |
| --- | --- | --- | --- | --- | --- |
| Male-headed HH | 0.231*** | -0.297** | -0.213 | 0.305*** | 0.021*** |
|  | (0.049) | (0.138) | (0.217) | (0.091) | (0.006) |
| Age of HH head | -0.003 | -0.002 | 0.009** | -0.015** | -0.009 |
|  | (0.004) | (0.006) | (0.004) | (0.007) | (0.008) |
| Occup in Agri | 0.297*** | 0.205** | 0.156*** | -0.109 | -0.224 |
|  | (0.075) | (0.098) | (0.051) | (0.166) | (0.219) |
| Edu: Primary | 0.221 | 0.186 | 0.203** | -0.172 | -0.255 |
|  | (0.239) | (0.188) | (0.098) | (0.215) | (0.303) |
| Edu: Secondary | 0.199*** | 0.247** | 0.207 | 0.295** | -0.216 |
|  | (0.063) | (0.118) | (0.223) | (0.139) | (0.286) |
| Edu: Higher | -0.203*** | 0.231*** | -0.390*** | 0.356*** | 0.108** |
|  | (0.061) | (0.072) | (0.124) | (0.115) | (0.047) |
| Labor | 0.087*** | -0.046 | 0.053 | -0.141*** | 0.063 |
|  | (0.026) | (0.054) | (0.051) | (0.038) | (0.065) |
| Land | 0.119** | -0.301*** | -1.102*** | 0.089 | -0.098 |
|  | (0.051) | (0.095) | (0.310) | (0.083) | (0.105) |
| Livestock | 0.136*** | 0.206*** | -0.099 | -0.106 | 0.081 |
|  | (0.042) | (0.051) | (0.098) | (0.114) | (0.083) |
| Asset | 0.234*** | -0.345*** | -0.301** | 0.170** | 0.102 |
|  | (0.072) | (0.097) | (0.142) | (0.079) | (0.100) |
| Credit | -0.161** | 0.227** | 0.173 | -0.298*** | -0.211 |
|  | (0.078) | (0.105) | (0.172) | (0.087) | (0.218) |
| Membership | 0.305*** | 0.301** | -0.165 | 0.161** | 0.199** |
|  | (0.097) | (0.147) | (0.163) | (0.072) | (0.095) |
| Market | 0.050*** | -0.056 | 0.038 | 0.041*** | -0.153*** |
|  | (0.015) | (0.055) | (0.041) | (0.013) | (0.046) |
| Extension | 0.043*** | -0.020 | 0.061 | 0.045** | 0.065** |
|  | (0.012) | (0.019) | (0.063) | (0.021) | (0.030) |
| Nepal dummy^#^ | 0.051*** | -0.029** | -0.065*** | -0.022 | -0.123** |
|  | (0.015) | (0.014) | (0.0202) | (0.019) | (0.059) |
| India dummy^#^ | -0.290 | 0.025 | 0.045* | 0.044 | 0.161*** |
|  | (0.285) | (0.034) | (0.025) | (0.051) | (0.042) |
| Kenya dummy^#^ | 0.233*** | -0.070*** | 0.092*** | -0.069*** | -0.295*** |
|  | (0.065) | (0.023) | (0.030) | (0.021) | (0.083) |
| Ethiopia dummy^#^ | -0.149*** | -0.015** | -0.020** | -0.033*** | -0.315*** |
|  | (0.043) | (0.007) | (0.009) | (0.011) | (0.072) |
| Food secure | 0.073*** | -0.153 | -0.196*** | -0.158 | 0.315 |
|  | (0.021) | (0.182) | (0.054) | (0.197) | (0.384) |
| Residual_foodsecure | 0.131 | 0.087 | 0.102 | 0.173 | 0.309 |
|  | (0.145) | (0.096) | (0.214) | (0.412) | (0.325) |
| Training | 0.210*** | 0.346 | -0.118* | 0.131** | 0.032** |
|  | (0.069) | (0.403) | (0.061) | (0.057) | (0.015) |
| Residual_training | 0.203 | 0.136 | 0.244 | 0.089 | 0.128 |
|  | (0.199) | (0.101) | (0.285) | (0.115) | (0.132) |
| Constant | -0.901*** | -1.214*** | -1.902*** | -2.109*** | -0.956*** |
|  | (0.282) | (0.315) | (0.522) | (0.601) | (0.298) |
| No. of observation | 4724 | 4724 | 4724 | 4724 | 4724 |

Log likelihood = -3275.15; Wald chi2(110) = 1335.81; Prob > chi2 = 0.0000

Notes:

1. *, **, *** refer to 10, 5, and 1% level of significance, respectively. Standard errors are in parentheses.
2. Variables ‘residual_food secure’ and ‘residual_training’ refers to residual term obtained from probit models estimated for food secure and training variables.

As none of the residual variables are significant, we have no serious endogeneity problem in our estimation. Based on this, we presented the results from models without residual terms. However, we presented the estimations for each individual country in the main results because it presents the analysis with differences in their adaptation strategies.

**References**

Abdulai, A., & Huffman, W. (2014). The Adoption and Impact of Soil and Water Conservation Technology: An Endogenous Switching Regression Application. Land Economics, 90(1), 26–43. <https://doi.org/10.3368/le.90.1.26>

Aryal, J.P., Rahut, D.B., Maharjan, S., Erenstein, O., 2018c. Factors affecting the adoption of multiple climate-smart agricultural practices in the Indo-Gangetic Plains of India. Natural Resources Forum 42, 141-158. <https://doi.org/10.1111/1477-8947.12152>
